# Combining brain perturbation and neuroimaging in non-human primates

**DOI:** 10.1016/j.neuroimage.2021.118017

**Published:** 2021-03-29

**Authors:** P. Christiaan Klink, Jean-François Aubry, Vincent P. Ferrera, Andrew S. Fox, Sean Froudist-Walsh, Béchir Jarraya, Elisa E. Konofagou, Richard J. Krauzlis, Adam Messinger, Anna S. Mitchell, Michael Ortiz-Rios, Hiroyuki Oya, Angela C. Roberts, Anna Wang Roe, Matthew F.S. Rushworth, Jérôme Sallet, Michael Christoph Schmid, Charles E. Schroeder, Jordy Tasserie, Doris Y. Tsao, Lynn Uhrig, Wim Vanduffel, Melanie Wilke, Igor Kagan, Christopher I. Petkov

**Affiliations:** a Department of Vision & Cognition, Netherlands Institute for Neuroscience, Meibergdreef 47, 1105 BA Amsterdam, the Netherlands; b Physics for Medicine Paris, Inserm U1273, CNRS UMR 8063, ESPCI Paris, PSL University, Paris, France; c Department of Neuroscience & Department of Psychiatry, Columbia University Medical Center, New York, NY, USA; d Department of Psychology & California National Primate Research Center, University of California, Davis, CA, USA; e Center for Neural Science, New York University, New York, NY, USA; f NeuroSpin, Commissariat à l’Énergie Atomique et aux Énergies Alternatives (CEA), Institut National de la Santé et de la Recherche Médicale (INSERM), Cognitive Neuroimaging Unit, Université Paris-Saclay, France; g Foch Hospital, UVSQ, Suresnes, France; h Ultrasound and Elasticity Imaging Laboratory, Department of Biomedical Engineering, Columbia University, New York, NY, USA; i Laboratory of Sensorimotor Research, National Eye Institute, Bethesda, MD, USA; j Laboratory of Brain and Cognition, National Institute of Mental Health, Bethesda, MD, USA; k Department of Experimental Psychology, Oxford University, Oxford, United Kingdom; J Newcastle University Medical School, Newcastle upon Tyne NE1 7RU, United Kingdom; m German Primate Center, Leibniz Institute for Primate Research, Kellnerweg 4, 37077 Göttingen, Germany; n Iowa Neuroscience Institute, Carver College of Medicine, University of Iowa, Iowa City, IA, USA; o Department of Physiology, Development and Neuroscience, Cambridge University, Cambridge, United Kingdom; p Interdisciplinary Institute of Neuroscience and Technology, School of Medicine, Zhejiang University, Hangzhou 310029, China; q Univ Lyon, Université Lyon 1, Inserm, Stem Cell and Brain Research Institute, U1208 Bron, France; r Faculty of Science and Medicine, University of Fribourg, Chemin du Musée 5, CH-1700 Fribourg, Switzerland; s Nathan Kline Institute, Orangeburg, NY, USA; t Division of Biology and Biological Engineering Tianqiao and Chrissy Chen Institute for Neuroscience; Howard Hughes Medical Institute; Computation and Neural Systems, Caltech, Pasadena, CA, USA; u Laboratory for Neuro- and Psychophysiology, Neurosciences Department, KU Leuven Medical School, Leuven, Belgium; v Department of Cognitive Neurology, University Medicine Göttingen, Göttingen, Germany; w Zuckerman Mind Brain Behavior Institute, Columbia University, New York, NY, USA; x Department of Radiology, Columbia University, New York, NY, USA; y Wellcome Centre for Integrative Neuroimaging, Department of Experimental Psychology, University of Oxford, Oxford, United Kingdom; z Leuven Brain Institute, KU Leuven, Leuven Belgium; Harvard Medical School, Boston, MA, USA; aa Massachusetts General Hospital, Martinos Center for Biomedical Imaging, Charlestown, MA, USA; bb Department of Neurosurgery, University of Iowa, Iowa city, IA, USA; cc Columbia University, New York, NY, USA

**Keywords:** Microstimulation, Optogenetics, Chemogenetics, Ultrasound, Lesion, Infrared, Causality, fMRI, Primates

## Abstract

Brain perturbation studies allow detailed causal inferences of behavioral and neural processes. Because the combination of brain perturbation methods and neural measurement techniques is inherently challenging, research in humans has predominantly focused on non-invasive, indirect brain perturbations, or neurological lesion studies. Non-human primates have been indispensable as a neurobiological system that is highly similar to humans while simultaneously being more experimentally tractable, allowing visualization of the functional and structural impact of systematic brain perturbation. This review considers the state of the art in non-human primate brain perturbation with a focus on approaches that can be combined with neuroimaging. We consider both non-reversible (lesions) and reversible or temporary perturbations such as electrical, pharmacological, optical, optogenetic, chemogenetic, pathway-selective, and ultrasound based interference methods. Method-specific considerations from the research and development community are offered to facilitate research in this field and support further innovations. We conclude by identifying novel avenues for further research and innovation and by highlighting the clinical translational potential of the methods.

## 1 Introduction

The brain is a complex dynamical network and an advanced understanding of its functional mechanisms requires both observational and perturbation studies. Observational studies aim to understand neural systems within a particular context. They are typically descriptive and correlative. Brain perturbation studies on the other hand can provide insights on cause and effect. By manipulating a neural substrate and observing the consequences of perturbation on relevant output measures (e.g., behavior, or neural activity patterns) one can demonstrate the substrate’s necessity and sufficiency for particular behaviors or cognitive functions (Krakauer et al., 2017; Marinescu et al., 2018). Neuroscience is rich with examples of observational studies leading to later experimental perturbations, from deducing the neurochemical properties of the giant squid action potential (Hodgkin and Huxley, 1952) to the recent successes of techniques like optogenetics, whereby neurons are transfected with light-sensitive channels that allow experimental control over action potentials by shining light of specific wavelengths onto the neurons (Deisseroth, 2015).

The field of neuroimaging is often criticized for its descriptive or correlational nature (Ramsey et al., 2010). In response, a range of analytical techniques have been developed as a proxy to derive causal inferences from otherwise correlational neuroimaging data. Examples of such approaches are dynamic causal modeling (DCM) for functional Magnetic Resonance Imaging studies (fMRI) (Friston et al., 2019, 2003) or Granger causality for electroencephalography (EEG) and magnetoencephalography (MEG) (Friston et al., 2012; Seth et al., 2015; Zhou et al., 2009). Despite an increase in the use of such analytical methods, they cannot replace the need for causal perturbation, and often require additional validation with empirical perturbation approaches. In humans, the combination of neuroimaging and transcranial magnetic stimulation (TMS) (Driver et al., 2009; Ruff et al., 2009), transcranial direct/alternating current stimulation (tDCS/tACS) (Cabral-Calderin et al., 2015; Saiote et al., 2013), or more recently transcranial focussed ultrasound stimulation (tFUS) (Lee et al., 2016a; Legon et al., 2018; Verhagen et al., 2019), has allowed non-invasive disruption or potentiation of neural processes with simultaneous monitoring of the effects of these perturbations on local and global brain activity patterns and behavior.

**Fig. 1:**
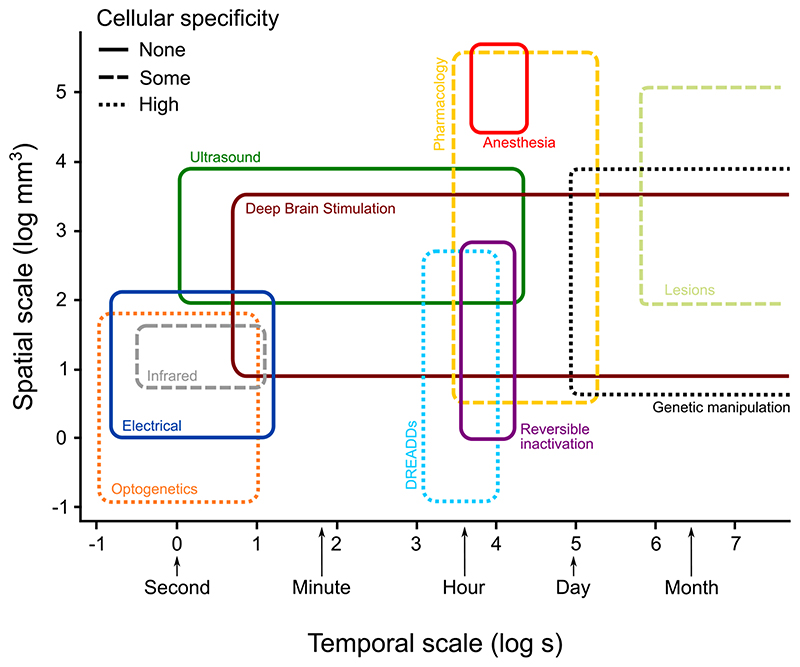
Schematic overview of the specificity of brain perturbation techniques used with neuroimaging in non-human primates. Different brain perturbation techniques operate on different spatial and temporal scales. Temporal scale is depicted on the horizontal axis (logarithmic; open ended). The range of temporal scales varies across techniques from subsecond time-scales to periods of months, or even years. The spatial scale of the perturbation methods is shown logarithmically on the vertical axis and ranges from tissue volumes smaller than a mm^3^ to having systemic effects. The spatial and temporal scales of individual methods are indicated with differently colored rectangles. Line styles indicate cellular specificity, with some techniques selectively perturbing brain activity in certain cell types and others lacking any cellular specificity. Note that pathway specific methods can add connectivity specificity to compatible perturbation techniques that have previously generally lacked such precision (see 9. Directional and pathway-selective approaches). Depiction style inspired by Grinvald and Hildesheim (2004) ind Sejnowski et al. (2014).

Brain perturbation techniques in both humans and animal models vary greatly in their scale and precision of effects (Fig. 1) with a tendency for non-invasive approaches to broadly affect many areas. All techniques furthermore come with their own advantages and limitations. On one side of this spectrum, inferences from neurological patient studies remain important, but such studies often have to deal with substantial lesion size and large variability in lesion location across patients. Pre-lesion experimental controls in the same individuals are almost always absent necessitating between-subjects comparisons with unaffected control participants. Some newer non-invasive approaches, like ultrasound stimulation, can provide more focal deep brain stimulation, and efforts are underway to extend their use from animal models to humans (Legon et al., 2020, 2014; Szablowski et al., 2018). All of the more precise perturbation methods, however, are invasive and as a consequence their application is restricted to animal models and, under restricted circumstances, human neurosurgery patients. The development of brain perturbation techniques for improved understanding of brain mechanisms, and novel diagnosis and treatment methods for human brain disorders thus typically involves foundational work in non-human animals.

Non-human primates (NHPs), in particular, remain indispensable in the translational pipeline from rodent models to humans (Mitchell et al., 2018; Roberts, 2020; Roelfsema and Treue, 2014), and the two most common neurobiological laboratory non-human primate models are the macaque and marmoset monkey. Because of the extensive similarities between humans and NHPs in terms of brain structure and cognitive functions (Hutchison et al., 2012a; Mantini et al., 2011; Neubert et al., 2015; Orban et al., 2006, 2004), NHP models are uniquely positioned to combine neural perturbation methods and neuroimaging to systematically investigate the structural and functional impact of brain perturbation and to solidify causal relationships between brain activity and behavior. With other animal models lacking either the cognitive capacity, or the structural and functional similarities to humans, and human research limited in the potential for detailed and systematic invasive neuronal recording and perturbation, the NHP model is truly in a unique position to investigate primate brain mechanisms and their relation to behavior (Roelfsema and Treue, 2014).

We present an overview of the state of the art in NHP brain perturbation methods that can be combined with neuroimaging to allow direct visualization of brain-wide neural perturbation effects. The potential translation of knowledge and methods from NHPs to humans is also highlighted wherever currently relevant or foreseeable in the near future. We survey both established and novel non-reversible (lesions) and reversible perturbation approaches using electrical, pharmacological, optical, optogenetic, chemogenetic, pathway selective, or ultrasound brain perturbation approaches. Our main aim is to provide a resource on the more commonly used techniques and a few new ones with substantial promise. We also aim to appeal to the broader scientific community, including those just interested in a brief background and impressions from using the techniques and the translational potential for humans. Laboratories that are already using or hoping to use these approaches might benefit from some of the methodological and practical considerations we describe. In this way we hope to encourage further research and innovation. We recognize the inherently limited scope of any one paper and embed our efforts in the broader PRIMatE Data Exchange (PRIME-DE) initiative (Milham et al., 2018). The PRIME Resource Exchange platform (Messinger et al., 2021), another PRIME-DE initiative, will furthermore support the resource and information exchange on brain perturbation approaches and neuroimaging in a dynamical community-driven way.

### 1.1 General considerations

Non-human primate neuroimaging is a complicated endeavour with substantial logistical and procedural demands (Chen et al., 2012; Farivar and Vanduffel, 2014; Goense et al., 2010; Logothetis et al., 1999). Combining imaging with brain perturbation introduces additional complexity to the experimental toolbox, and while some of these additional complexities are technique-specific, other considerations are more generic and apply to some degree to all perturbation techniques.

A first issue to consider is whether a study will be performed in anesthetized or awake animals. This choice will primarily depend on the specific research question and the planned imaging modality. Anesthetized and awake primate neuroimaging each come with their own procedural aspects, which are addressed in detail elsewhere (Basso et al., 2021). Studies involving anesthetized subjects are often important for initial development during which the fine-tuning of equipment might take a substantial amount of time and the animal is not required to be conscious. However, if the influence of brain perturbation on both brain activity and self-initiated behavior (e.g., perception, cognition, movement) is investigated, the subject needs to be awake to perform such behaviors. Even if there is no necessity for behavioral output, it is still beneficial to perform a study in awake animals because anesthetic agents tend to affect various physiological processes, such as dampening activity in several cortical and subcortical areas. Due to these physiological side effects, it can often be beneficial to directly compare the impact of perturbation in anesthetized and awake animals. For instance, the stimulation strength thresholds at which measurable brain activations are evoked may be significantly higher in anesthetized animals compared to awake ones (Murris et al., 2020; Premereur et al., 2015). Moreover, both the pattern and amplitude of perturbation-induced activity changes can vary as a function of brain state (Moeller et al., 2009 Murris et al., 2020; Petkov et al., 2015 Premereur et al., 2015; Rocchi et al., 2021).

Another crucial decision that needs to be made in planning the perturbation study is what kind of neuroimaging signal will be measured. For NHP MRI, for instance, the two most common fMRI measures are the Brain Oxygen Level Dependent (BOLD) signal and contrast-agent (e.g. MION) enhanced fMRI (Leite et al., 2002; Vanduffel et al., 2001). Both are indirect measures of neuronal activity and while the two generally reflect similar signal fluctuations with different signal-to-noise ratios, there are also differences. These differences are generally attributed to neurovascular coupling effects, or the intrinsic relation between neuronal activity and the respective imaging signals (Logothetis, 2008; Smirnakis et al., 2007). Other approaches, such as Positron Emission Tomography (PET), will have their own considerations. Perturbation-induced neuronal activity modulation could potentially be different for PET, BOLD and cerebral blood volume (CBV) signals. Another consideration is that brain perturbation may cause vascular effects in addition or instead of neuronal effects, which are difficult to dissociate from neural effects with fMRI (Choi et al., 2006).

Once a decision has been made on what perturbation technique will be used, what neuroimaging signal will be measured, and whether the subject will be anesthetized or awake and potentially behaving, there are a number of practical issues in the execution of the planned experiment that are common among many perturbation techniques. Most importantly, how do we make sure that the correct brain area is targeted so that the perturbation is both strong enough to cause the desired effect and specific enough to avoid unintended effects (e.g., unintended involvement of neighboring areas). The exact solution will again depend on the perturbation method and brain area in question. The targeting process is commonly guided by, or confirmed with, neuroimaging, which allows visualisation of the perturbation effect, and in some cases also of the delivery device itself (see Box 1). Ideally, such localization methods are coupled with other read-outs such as electrophysiology, functional activity, or perturbation-induced behavior.

The incorporation of brain perturbation equipment in a spatially restricted imaging environment presents another general challenge.

Whether MRI or PET, there will likely be a restricted amount of space for equipment in the imaging scanners. The magnetic fields generated by the MRI scanner further limit the use of ferromagnetic materials, both for safety reasons and because the material may disrupt data acquisition. It is possible that the presence of perturbation equipment, or even the actual application of brain perturbation, will distort the neuroimaging signal and hinder the read-out of the neural effects of perturbation. One also needs to consider that interactions amongst the equipment, the static magnetic field, the changing magnetic fields, and RF-gradients, can induce currents and cause deviations in the applied perturbation beyond an experimenter’s control. Such deviations can have negative effects on the perturbation regime, data acquisition, neural tissue, and ultimately the scientific results. In the following technique-specific sections, we discuss several common and emerging types of perturbation methods that can be combined with neuroimaging. Specific methodological considerations are highlighted for each technique.

## 2 Permanent lesions and neuroimaging

### 2.1 Permanent lesions and fMRI

Early non-human primate lesion studies were directly motivated by attempts to understand the deficits of seminal neurological patients like Phineas Gage and Henry Molaison (H.M.), relating brain damage to the loss of certain cognitive functions. Reports of Gage’s impairments inspired investigations of the macaque frontal lobe. Lesions to the prefrontal cortex were subsequently shown to cause impairments to shortterm memory during distraction (Jacobsen, 1935). More precise lesion studies later localised these effects to the lateral prefrontal cortex, in the region of the principal sulcus (Blum, 1952; Butters and Pandya, 1969; Goldman and Rosvold, 1970; Mishkin, 1957). Although lesions are commonly associated with localizationist ideas, many researchers using the lesion approach are aware of the need to investigate connected areas and to control for the potential confound of affecting fibers of passage. The research scope thus expanded in an attempt to identify networks of interconnected regions that may contribute to various aspects of cognition.

After the pioneering studies of Brenda Milner and colleagues identified anterograde memory deficits in patient H.M. (Scoville and Milner, 1957), the task shifted to model and replicate H.M.’s deficits in non-human primates. Gaffan found that lesioning the fornix bundle (a major connection between the hippocampus and subcortical structures including the mammillary bodies, anterior thalamus and medial septum) caused severe memory deficits (Gaffan, 1974). A few years later, Mishkin found similarly profound memory deficits following lesions of the macaque hippocampus, amygdala and surrounding structures (Mishkin, 1978). This inspired lesion studies in the non-human primate to identify the contributions of different cortical and subcortical structures to distinct aspects of memory function (Basile et al., 2020; Baxter and Murray, 2001; Froudist-Walsh et al., 2018b; Gaffan, 1994; Mitchell et al., 2008, 2007; Mitchell and Gaffan, 2008; Murray et al., 1998; Zola et al., 2000). Despite precise targeting of lesion sites in many non-human primate lesion studies, assessment relied primarily on behavior, making it difficult to identify the lesion’s neural impact and the brain’s recovery response (plasticity).

Functional MRI provides a dynamic view of activity throughout the brain and has been used to identify distributed networks underlying many cognitive functions in humans (Smith et al., 2009; Thomas Yeo et al., 2011) and macaques (Hutchison et al., 2012b). By combining focal lesions with whole-brain fMRI, researchers have now shown that focal lesions (e.g., to the hippocampus, neocortical regions, or white matter tracts) can have hugely distributed effects on connectivity in distant parts of the brain (Adam et al., 2020). Animal neuroimaging lesion studies allow pre- and post-lesion comparisons in the same animal as well as longitudinal assessment. This offers a level of precision and insight that cannot be obtained in humans, thus enhancing both the scientific knowledge gained from the lesion approach as well as the relevance and translational benefits for humans.

Humans can suffer permanent lesions from various causes (e.g. strokes, accidental injury, neurosurgery, traumatic brain injury etc). Studies to date have focused on changes in resting-state correlated activity (i.e., ‘functional connectivity’) following lesions, which can correlate with behavioral recovery following stroke lesions (He et al., 2007) or with structural connectivity changes in white matter tracts (Meng et al., 2018; Shamy et al., 2010). Clinical studies are now also documenting brain changes over time following permanent injury resulting in neglect or aphasia (Saur et al., 2006; Berthier et al., 2011; Hartwigsen et al., 2019; Stockert et al., 2020; Umarova et al., 2016). Responses to damage in the human brain are, however, rarely homogenous and vary substantially across subjects. Usually, baseline measures prior to the neurological event are lacking, which means that the estimation of behavioral deficits and changes to brain structure, connectivity and function rely on comparisons between patients and age-matched control subjects, or population average templates (Foulon et al., 2018; Salvalaggio et al., 2020). This is one of the reasons animal lesion studies remain indispensable as models for permanent brain injury in humans. Combined with neuroimaging, they can provide an important vantage point on the functional neural impact of the lesion and the brain’s long-term postinjury recovery response (plasticity). Such studies typically involve cytotoxic injections, aspiration or ablation to lesion the targeted brain structure. Cytotoxic injections focally destroy the cells of the target structure, while removal of the structure via aspiration or ablation also affects fibers of passage (white matter tracts) in or traversing through the target structure.

#### 2.1.1 Anatomical and functional imaging of lesion effects

**Fig. 2:**
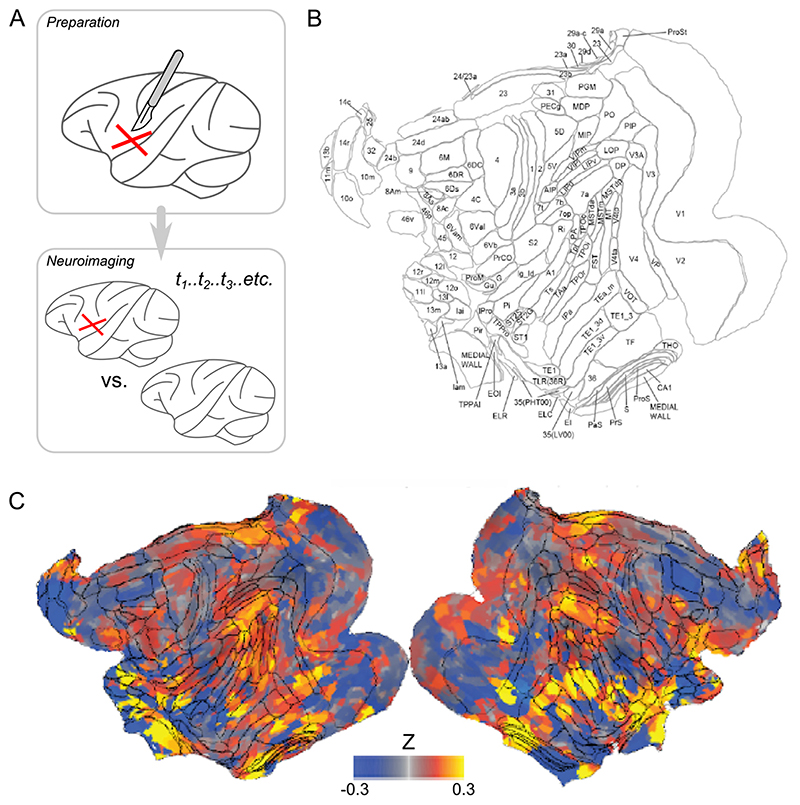
Permanent lesions and fMRI. A) In the preparation phase of an experiment, a pre-lesion scan (e.g. structural MRI, functional MRI, PET) is acquired. A lesion is then made in a specific area of the brain, e.g. by cutting a fiber bundle or injecting an excitotoxin such as NMDA. In the subsequent phase of the experiment further scans are acquired and lesioned animals are compared with control animals without a lesion and with their own pre-lesion scan. This comparison can be performed at different time-points (t1, t2, t3, etc) to investigate dynamic adaptive and maladaptive plasticity over weeks and months following the lesion. B) Anatomical regions from the LV-FOA-PHT composite cytoarchitectonic parcellation (Van Essen et al., 2012), as used in C) Whole-brain seed-to-voxel connectivity maps for an example seed in the right hemisphere orbitofrontal area 12 (ROI: 12o). Maps show changes in functional connectivity (correlation strength) after bilateral fornix transections (shown schematically for one hemisphere in the bottom right inset). Adapted from Pelekanos et al. (2020).

Neuroimaging can be performed prior to, and after, the lesion(s) (Fig. 2 A) to visualize the loci and extent of permanent lesions as well as changes in: 1) functional connectivity within and/or between gray matter structures, 2) white matter connectivity (between gray matter structures), 3) volume or thickness of gray matter structures, 4) metabolic processes and 5) neural activity in response to stimuli or a task. By combining the precise localization of lesions using structural MRI with longitudinal fMRI assessment in the macaque, it is possible to tease apart the causal effects of different aspects of lesions (such as the size, location and timing) on the dynamics of whole-brain plasticity and behavior. MRI contrast agents (e.g. Gadolinium, see Box 1), injected intravenously, can enhance the detection and localization of brain lesions and breakdown of the brain-blood barrier. Diffusion-weighted MRI, e.g. ‘apparent diffusion coefficient’ (ADC) imaging that is unaffected by T2-weighting can facilitate the detection of edemas or necrosis due to infarction. Perfusion-weighted imaging (PWI) can be used to detect areas that are structurally intact but not receiving enough oxygen to function normally (i.e. the ischemic penumbra; Schlaug et al., 1999). These are areas that are non-functional, but likely treatable and that may recover over time with or without treatment (Balezeau et al., 2021). PWI is often performed using dynamic susceptibility contrast imaging with Gadolinium. Increasingly, arterial spin labeling (ASL) has been presented as an alternative that does not require an invasive contrast agent (Zaharchuk, 2014). This is gaining popularity in clinical studies, and the acquisition of such data may be important to maximise the translational potential of non-human primate studies, particularly stroke models.

#### 2.1.2 Connectivity impact by lesions

The corpus callosum is the largest white matter tract in the brain, and it directly connects interhemispheric homotopic, as well as some non-homotopic, regions. Surprisingly, however, in macaque monkeys, sectioning the corpus callosum alone has relatively subtle effects on interhemispheric functional connectivity (O’Reilly et al., 2013), suggesting that functional connectivity between the hemispheres can be maintained via secondary routes. Indeed, when the smaller anterior commissure bundle was also sectioned, interhemispheric functional connectivity was drastically reduced.

Interhemispheric reorganisation was also observed in a longitudinal study of two monkeys with lesions to the dorsolateral prefrontal cortex (dlPFC) (Ainsworth et al., 2018). After a unilateral lesion to the left principal sulcus, functional connectivity of frontal regions was transiently disrupted, before restoration of close-to-normal connectivity 8weeks post-damage. However, after a further unilateral lesion to the principal sulcus in the opposite hemisphere, functional connectivity in frontal regions was permanently disrupted, with a concurrent increase in fronto-parietal connectivity, which may represent compensatory plasticity (Ainsworth et al., 2018). The lesions furthermore affected visuospatial and non-spatial working memory, with some recovery of function that may reflect the observed changes in functional connectivity. Adam et al. (2020) also investigated the effects of lesions to the right hemisphere principal sulcus and adjacent frontal eye fields using a free-choice saccade task. After lesioning, monkeys displayed spatial deficits similar to those seen in humans following damage to regions of the dorsal attention network. In particular, monkeys transiently exhibited visuospatial neglect, before transitioning to a milder impairment (contralesional visual extinction), which in turn mostly normalised by the end of the study (Adam et al., 2019). They observed that the pattern of functional connectivity changes depended on the size of the lesion. Monkeys with smaller lesions had transient increases in functional connectivity throughout the frontoparietal network, before returning to baseline levels. In contrast, monkeys with larger dlPFC lesions showed sustained increases in functional connectivity over the entire study period (Adam et al., 2020). This study exemplifies the value of testing monkeys at multiple timepoints pre- and post-lesion in order to better characterise the dynamic trajectory of post-lesion recovery. Brain plasticity in acute and chronic post-lesion stages can, however, be strikingly different (Berthier et al., 2011; Saur et al., 2006), suggesting that distinct mechanisms could underlie acute, chronic and very late recovery phases (Hillis, 2006; Hillis and Heidler, 2002).

#### 2.1.3 Insights on mechanisms of plasticity

Non-human primate studies combining lesions with fMRI can also provide insight into the cellular mechanisms that govern dynamic post-lesion plasticity across the brain. After bilateral hippocampal lesions, monkeys are impaired at recalling recent and remote visuospatial discriminations in an object-in-place learning task, but they can still learn new visuospatial discriminations (Froudist-Walsh et al., 2018b). Resting-state fMRI scans at the pre-lesion stage, and at 3 and 12 months post-lesion, provided insight into the acute (<3 months) and chronic (3-12 months) stages of post-lesion brain plasticity. Pre-lesion patterns of connectivity (defined either using functional connectivity or invasive tract-tracing) and the density of neuronal and non-neuronal cells predicted how surviving cortical regions would adapt their connectivity in the acute and chronic post-lesion stages (Froudist-Walsh et al., 2018a). In particular, areas that acted as interconnected hubs in the pre-lesion network had sustained reductions in functional connectivity, whereas areas with higher density of non-neuronal cells (presumably glial) increased their network participation through increased activity and interconnectivity in the chronic stage. Cortical areas that were strongly connected to the hippocampus before the lesion adapted to an acute loss of connectivity to distant regions by increasing their connectivity with local regions in the later chronic stage. This highlights that the effects of lesions are dynamic and that long-term effects depend on plastic changes of cellular constituents and connectivity that can now be visualized.

Another study in monkeys investigated resting state functional and structural changes as a consequence of learning to make complex vi- suospatial discriminations, before and after fornix transection (a permanent subcortical white matter tract lesion that impairs learning of visuospatial discriminations) (Pelekanos et al., 2020). After learning, changes were observed in functional connectivity of gray matter structures in the frontal and cingulate cortex, inferotemporal cortex, subicular complex, and the dorsal medial thalamus interconnected with these cortical regions (Fig. 2 B and C). Structural changes were also observed in white matter tracts connecting these regions, including the ventral prefrontal tract, uncinate fasciculus, and fornix. After the permanent lesion, learning was affected, and structural connectivity was altered in the ventral prefrontal tract, but not in the uncinate fasciculus. Functional connectivity in the contiguous gray matter structures was also changed. This work captures the importance of both cortico-cortical and thalamo-cortical interactions in reward-guided learning in the normal primate brain and allows for the identification of brain structures important for memory capabilities within the same animals after permanent brain lesions (Pelekanos et al., 2020).

Combining behavioral measures with neuroimaging in awake monkeys, Hadj-Bouziane and colleagues first identified anterior and posterior face-selective areas in the inferotemporal cortex, as well as areas that respond more to emotional facial expressions than to neutral ones (Hadj-Bouziane et al., 2012). They then investigated changes in brain function after excitotoxic lesions to the amygdala with fMRI, while the monkeys viewed different facial expressions. After the amygdala lesions, inferotemporal regions that had previously responded preferentially to certain emotional facial expressions no longer responded differentially, despite having a preserved preference for faces compared with scrambled faces or non-face objects. This study was thus able to identify changes in functional activity and tuning in one brain area following lesions in another.

#### 2.1.4 Developmental timing of lesions

Lesions to the hippocampus that occur in the neonatal period have persistent effects on the functional connectivity of the dlPFC in adult monkeys (Meng et al., 2014). This finding provided an important translational link between an influential rodent neonatal ventral hippocampal lesion model of schizophrenia (Tseng et al., 2009) and findings of reduced dlPFC functional connectivity with regions such as the hippocampus in patients with schizophrenia (Pettersson-Yeo et al., 2011). The pattern of functional connectivity changes following a neonatal hippocampal lesion appears to differ from those seen in monkeys that receive hippocampal lesions in adulthood (described above), suggesting an interaction between post-lesion plasticity and developmental plasticity. The importance of lesion timing is also demonstrated by lesions of visual cortical area V1. Following aspiration lesions of V1 in the adult monkey, visually driven BOLD responses in connected regions are reduced by up to 70% (Schmid et al., 2009). V1 itself shows limited reorganisation following lesions to the retina in adult monkeys (Smirnakis et al., 2005). However, after naturally occurring neonatal lesions of V1, the extrastriate visual network connectivity was largely intact (Bridge et al., 2019). The time-course of lesion impact on network reorganisation and behavior could be more effectively demonstrated in future studies by investigating the effects of similar lesions at different developmental stages and as a function of aging.

### 2.2 Discussion and outlook

The combination of lesions and fMRI in non-human primates has been crucial in identifying and distinguishing the effects of lesion location, size and timing on whole-brain changes in connectivity, behavior, and cognitive capacity. These factors are difficult to precisely control in studies of human patients. The NHP neuroimaging evidence after permanent lesions can thus provide unique translation of information to humans for developing more effective diagnosis, prognosis and treatment options after permanent brain injury. The combination of these techniques with invasive and post-mortem anatomy has also begun to reveal cellular mechanisms underlying post-lesion recovery dynamics. These studies can be used to inform and test computational models of lesion effects and post-lesion plasticity, which can in turn guide future experiments (Froudist-Walsh et al., 2020; Kaiser, 2020). Going forward, non-human primate studies offer a unique method to investigate links between cellular and synaptic plasticity mechanisms after permanent lesions and subsequent large-scale changes to distributed brain network activity underlying higher cognitive functions. To-date, studies have primarily examined the effects of lesions on changes in anaesthetised resting-state functional connectivity. The utility of combined non-human primate lesion and fMRI studies for understanding compensatory mechanisms could be increased by further studying lesion effects on changes in task-related fMRI activation in behaving primates. This is particularly true for tasks that assess higher cognitive functions, for which non-human primates are the ideal animal model. Efforts to further develop non-human primate task-based fMRI are ongoing (Milham et al., 2020). Lesion-fMRI has been used to identify potential sites of compensatory plasticity. These claims can be explicitly tested in the future by combining lesion-fMRI with other perturbation methods described below. By altering nodes in the post-lesion network, researchers will be able to identify which nodes contribute to compensation (useful plasticity) or further disruption of function (maladaptive plasticity). Lesion-fMRI studies in non-human primates also carry substantial potential for testing novel treatments that promote compensatory plasticity and improve neural and behavioral recovery after lesions, an important translation to clinical scenarios. Future work using systemic pharmacological or focal perturbation methods in the lesioned brain can chart a clear translational path towards imaging-guided treatments to promote compensatory plasticity following permanent brain injury.

## 3 Reversible lesions and neuroimaging

### 3.1 Reversible pharmacological inactivation and fMRI

**Fig. 3:**
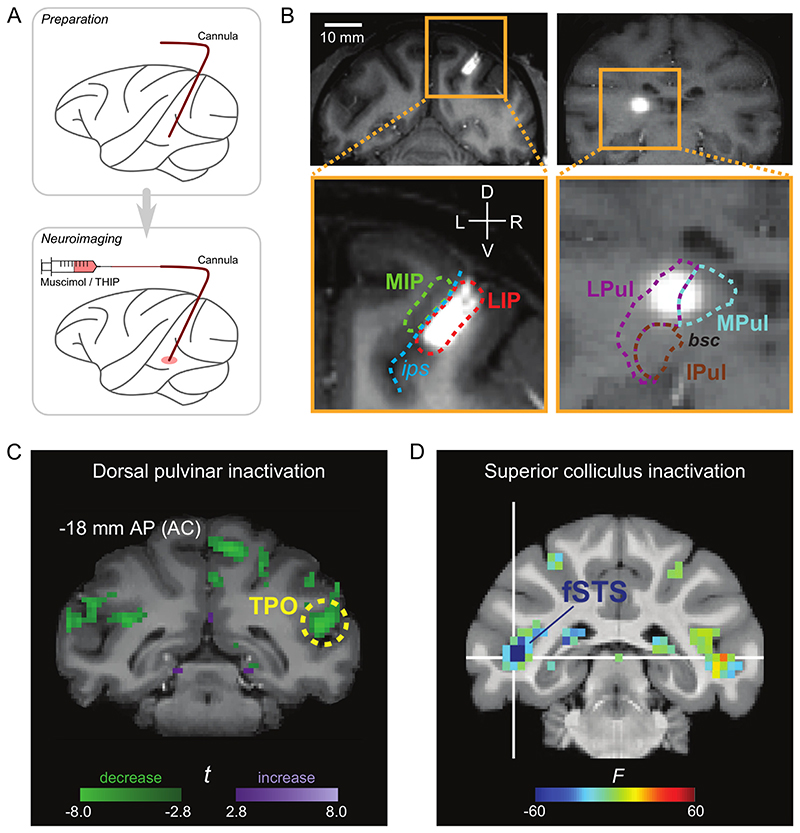
Reversible pharmacological lesions and neuroimaging. A) Schematic of the technique. A cannula is implanted before the neuroimaging stage of the experiment so that pharmacological agents such as muscimol or THIP can be administered while the animal is in the scanner. B) Coronal sections (T1-weighted) showing examples of (4 *μ*l) muscimol injection into the lateral intraparietal area LIP (left) and THIP injection into the dorsal pulvinar (right), together with a Gd contrast agent (1:100) (L/R: left/right; V/D: ventral/dorsal; LPul: lateral pulvinar; MPul: medial pulvinar; IPul: inferior pulvinar; bsc: the brachium of the superior colliculus). Adapted from Wilke et al. (2013, 2012). C) Reversible inactivation of the dorsal pulvinar leads to a bilateral decrease of the contralesional cue-evoked activity, particularly in area TPO in the superior temporal sulcus (STS) (Wilke et al., 2010a b). D) Reversible inactivation of the superior colliculus leads to a decrease in the attentional modulation of activity that is strongest in the fundus of the STS (fSTS) of the inactivated hemisphere. Adapted from Bogadhi et al. (2019)

Focal reversible lesion(s) and inactivation (Fig. 3 A) provide a complementary approach to permanent structural and cytotoxic lesions described above, with the advantage of this being short-term and repeatable with interleaved recovery periods. The approach has been a mainstay of behavioral study and is more recently also being used in combination with electrophysiological recordings in NHPs. So far, however, only a handful of fMRI studies have utilized reversible inactivation to investigate the neural effects of brain perturbation on interconnected brain regions during perceptual or cognitive tasks (Balan et al., 2019; Bogadhi et al., 2019; Schmid et al., 2010; Van Dromme et al., 2016; Wilke et al., 2012). The reversible inactivation method typically relies on local injections of a substance that inhibits neuronal activity, such as GABA-A receptor agonists muscimol (5-(Aminomethyl)-isoxazol-3-ol) or THIP (4,5,6,7-tetrahydroisoxazolo (5,4-c)pyridin-3(-ol)). The GABA-A agonists act at extrasynaptic receptors to inactivate neurons locally (i.e., at the soma and the dendrite) and do not affect fibers of passage, unlike other drugs such as TTX and lidocaine (Waszczak et al., 1980). THIP has substantially lower affinity and binding rate to GABA-A receptors compared to muscimol (Jones and Balster, 1998; Waszczak et al., 1980), and is therefore particularly useful when larger areas need to be inactivated and when only modest behavioral deficits are desired (Wilke et al., 2010a,b).

#### 3.1.1 Cortical and subcortical insights from combined pharmacological inactivation and fMRI

Two fMRI studies inactivated the lateral intraparietal region (area LIP) with muscimol to study the neural basis of spatial decision, attention and neglect-like symptoms, and their compensation (Balan et al., 2019; Wilke et al., 2012). Wilke and colleagues employed a time-resolved event-related design using a delayed memory saccade task with instructed and free choice targets, showing that a unilateral LIP lesion leads to decreased BOLD activity for contralesional single instructed targets, most pronounced in the upper bank of the superior temporal sulcus. Furthermore, in choice trials where contralesional targets were chosen despite an overall inactivation-induced ipsilesional bias, several frontal and parieto-temporal areas in both hemispheres showed a putatively compensatory activity increase (Wilke et al., 2012). Similarly, Balan and colleagues, employing covert attentional search and detection tasks, demonstrated that unilateral LIP inactivation leads to fast, widespread and largely activity-enhancing changes in interconnected attention-related regions in both hemispheres, that were most pronounced in area FEF (Balan et al., 2019). Stimulus competition and attentional selection mechanisms spanning the entire visual field are common aspects of the tasks in the two studies. The compensatory recruitment of healthy nodes within and across hemispheres is in agreement with a recent permanent lesion study of dlPFC showing that alleviation of lesion-induced spatial deficits is associated with increased resting-state functional connectivity between the ipsi-lesional parietal cortex and the contralesional dlPFC (Adam et al., 2020; see Section 2). Hence, reversible and permanent lesions provide complementary evidence for the recruitment of the bilateral frontoparietal network during recovery, at shorter and longer timescales.

Van Dromme and colleagues inactivated a caudal subregion of the intraparietal sulcus (CIP) with muscimol to study its contribution to 3D object processing (Van Dromme et al., 2016). Using fMRI, they first identified several brain regions including CIP that were more activated by curved stimuli than by flat stimuli. They then showed that the unilateral inactivation of CIP caused perceptual deficits in a depth-structure discrimination task and that BOLD activity during passive viewing was reduced not only in the adjacent parietal region (AIP) but also in the distant anterior inferotemporal cortex in both hemispheres (Van Dromme et al., 2016).

Causal mechanisms of spatial perception, attention and decision making have also been studied in subcortical structures. Combining structural lesions in V1 with reversible THIP inactivation of the lateral geniculate nucleus (LGN), Schmid and colleagues used fMRI to show that LGN input to extrastriate cortex is crucial for blindsight when V1 is lesioned (Schmid et al., 2010). Similar to the parietal inactivation results (Wilke et al., 2012), inactivation of the thalamic dorsal pulvinar (dPul) with THIP was shown to lead to contralesional choice deficits during memory saccades (Wilke et al., 2013). This was coupled with a widespread decrease in BOLD responses to contralesional saccade targets in both hemispheres that was most pronounced in the upper bank of the superior temporal sulcus, and a relative enhancement of activity in visuomotor regions associated with contralesional decisions during choice trials (Wilke et al., 2010a,b) (Fig. 3B and C).

Bogadhi and colleagues used fMRI combined with microinjections of muscimol into the intermediate layers of the superior colliculus (SC) to investigate how the SC contributes to the control of visual selective attention (Bogadhi et al., 2019). Previous work had shown that reversible unilateral inactivation of the SC produces major deficits in visual selective attention akin to visual neglect (Lovejoy and Krauzlis, 2010) but without changing attention-related modulations in the extrastriate visual areas that process the visual feature signals needed for the task (Zenon and Krauzlis, 2012). This combination of results was puzzling, because extrastriate visual areas have long been implicated as the primary site of attention modulation. By using fMRI to quantify attention-related modulation across cortex before and during muscimol inactivation of the SC, Bogadhi and colleagues identified an area in the superior temporal sulcus as the cortical region showing the largest loss of attention-related modulation during the attention deficits caused by SC inactivation (Fig. 3 D). This illustrates the value of combining fMRI with causal manipulations to obtain a broad assay of task-specific functional connectivity. Unlike areas in the frontal and parietal cortex that have long been known to be involved in visual selective attention, the significant role of the superior temporal sulcus in the control of attention has been recently demonstrated through fMRI (Bogadhi et al., 2018; Caspari et al., 2015; Patel et al., 2015; Stemmann and Freiwald, 2019; Wardak et al., 2010). Taken together, the studies of the nodes of the frontoparietal and subcortical circuitry supporting attention and goal-directed action - LIP, dlPFC, SC and dPul - demonstrate 1) the importance of rapid bi-hemispheric activity changes in remote brain regions, posing a challenge for the interhemispheric rivalry theory of spatial processing and neglect; 2) the high dependence of deficit-associated neural changes on task demands and behavioral context; and 3) the significance of the superior temporal sulcus in spatial attention/target selection and neglect-like symptoms such as spatial action bias and extinction.

Local inactivation is also a powerful technique to study functional connectivity. For instance, Turchi and colleagues injected muscimol into two distinct subregions of the nucleus basalis of Meynert (NBM), which provides strong neuromodulatory cholinergic and GABAergic inputs to cortex and subcortical structures such as hippocampus and amygdala (Turchi et al., 2018). They found a suppression of shared, or global, signal components of cortical fluctuations ipsilateral to the injection. Despite these global signal reductions, major known restingstate networks maintained their spatial structure, suggesting the contribution of NBM to the global component of resting-state functional connectivity.

#### 3.1.2 fMRI-targeted reversible inactivation

In addition to local inactivation-fMRI studies that investigated distributed neural effects of the inactivation, several studies utilized task-related fMRI to target specific inactivation loci based on the presence of activity correlated with behavior. Sadagopan and colleagues used fMRI-guided localization of a highly selective middle-lateral face patch to show that silencing it with muscimol impairs face detection in natural visual scenes (Sadagopan et al., 2017). In a series of demanding cognitive experiments, Miyamoto and colleagues used fMRI to identify brain regions involved in metacognition, and confirmed their functional contribution with muscimol inactivation. More specifically, using fMRI, metamemory (self-monitoring and confidence evaluation of one’s own memory content) was localized to prefrontal area 9 (or 9/46d) for temporally remote events, and to area 6 for recent events (Miyamoto et al., 2017), while inactivation of frontopolar area 10 selectively impaired confidence judgment of non-experienced events (Miyamoto et al., 2018).

### 3.2 Methodological considerations

#### 3.2.1 Drug delivery options

There are two main ways to deliver a drug to the target brain region. One approach is to use an implanted MRI-compatible (PEEK or fused silica) cannula through which another thinner injection cannula is acutely inserted to the target (Schmid et al., 2010; Wilke et al., 2012). Injections can also be done outside of the scanner using a metal cannula or an injectrode (a recording electrode paired with the injection channel). This is typically done with a recording chamber and positioning grid, using a micro-drive for gradual insertion. The mode of drug delivery could be relevant for the experimental design. For instance, using a fully MRI-compatible setup with an implanted cannula, pre-injection baseline runs can be collected while the animal is already in the scanner, before and after the injection in the same session, allowing for easier paired statistical analysis. Most experimental designs rely on separate baseline and injection sessions, for which the injection approach described below might be as efficient.

#### 3.2.2 Avoiding imaging artifacts

In order to avoid imaging artifacts that might be introduced by the presence of the injection cannula and other experimental equipment, it is possible to inject the pharmacological agent while the subject is outside the fMRI facility, and then remove the hardware before the imaging session. This requires careful timing, and the ability to perform the injection at a location a short distance from the magnet. For example, for injections into the SC, small volumes of muscimol (about 0.5 *μ*l) can be injected over a period of about 20 minutes, then some additional time (~20 minutes) is necessary for the muscimol to diffuse before the removal of the cannula. The delayed removal of the cannula is important to prevent the muscimol from ascending along the penetration track as the cannula is retracted. Once the cannula and mounting hardware are removed, the chamber can be filled with a gel that minimizes the distortion at the surface of the brain, and imaging can be done while the muscimol continues to suppress neuronal activity.

#### 3.2.3 Control condition design

Muscimol injections into the SC typically involve concentrations of 5 mg/ml and injection volumes of less than 1 *μ*l (usually closer to 0.5 *μ*l). The small volumes are appropriate given the small size of the SC (a few mm across) and avoid problems that can arise if muscimol spreads outside the SC into adjacent structures. Inactivation of the SC produces well-established changes in the metrics of saccadic eye movements (Lee et al., 1988) that provide a very useful positive control for the effectiveness of the injection. For example, when testing the effects of SC inactivation on attention-related BOLD modulation, the subject can perform a short block of saccadic eye movements, in between each of the blocks of the main task, to determine whether or not the drug injection is still effective (Bogadhi et al., 2019). Given that the effects of a single injection will peak and then decline over time, it is extremely helpful to have this type of positive control to test whether or not the pharmacological manipulation was successful during each imaging block. Alternatively, in some experiments the main task can serve as a behavioral readout for the inactivation time course, e.g. the contralesional/ipsilesional choice proportion in free-choice trials is a sensitive measure of the inactivation in LIP and dPul.

Typically, inactivation effects are observable from 30 to 60 min after injection, peak at 1.5–2 h and last a few hours. Some studies however report a much later peak at 18 h after the injection (Liu and Pack, 2017). The exact time course might depend on the concentration (typically 3–10 mg/ml), the injection volume (typically 0.5–5 *μ*l), the drug (muscimol or THIP), and the physiological properties of the target structure. For these reasons, brain physiology might not necessarily have returned to baseline the day after the injection, which is something to keep in mind when recording control data without inactivation injections. While saline sham injections are an accepted control for the specificity of the pharmacological drug effect, every penetration and injection can potentially cause some tissue damage along the track and inside the target structure. Therefore, it might be advisable to compare a few saline injection sessions with the sessions without any injection. If there are no observable differences in behavior or neural activation patterns between the saline and no-injection sessions, no-injection sessions can be used as baseline controls.

#### 3.2.4 Risks and solutions

There are three main risks associated with reversible inactivation experiments. As with any intracranial procedure, the penetration and drug injection can introduce infections. To minimize this risk, the procedure should be as sterile as possible, and the injection solution should be based on a sterile PBS buffer or be sterile-filtered. The insertion of the injection cannula can cause bleeding if vessels are damaged, although MRI can both help to avoid large vessels and monitor the status of (recovery from) any bleeding that might still occur (Balezeau et al., 2021). Lastly, for certain deep structures, e.g. LGN, SC, pulvinar, and caudate, the nearby ventricles present a special risk because the injection of a potent GABA-A agonist such as muscimol into a ventricle can cause drowsiness and potentially suppress respiration. Fortunately, the latter two risks can be mitigated with a careful pre-penetration targeting and vessel avoidance plan (see Box 1), and with concurrent visualization of the penetration track and injection site with an MR contrast agent prior to injection.

#### 3.2.5 Assessing the spatial extent of inactivation with co-injection of gadolinium

In several of the fMRI studies described above, the extent of inactivation was confirmed by co-injection of the MRI contrast agent gadolinium or manganese chloride (see Box 1). In prior work, MRI-visualized spread of the gadolinium contrast agent closely matched the muscimol distribution volume (Allen et al., 2008; Heiss et al., 2010). Although such labeling proved extremely valuable for guiding the analysis of inactivation effects, several issues need to be taken into account when evaluating drug spread with gadolinium. First, gadolinium passes through the extracellular space until it is absorbed by the blood stream via CSF. In contrast, GABA-A agonists such as muscimol/THIP pass through the extracellular space until they either bind to the GABA-A receptor on the surface of a neuron or are enzymatically degraded or absorbed into the bloodstream or CSF (Heiss et al., 2005). Second, while gadolinium travels along the axon, the myelinated fiber tracts represent a diffusion barrier for muscimol and the drug does not bind there (Allen et al., 2008; Heiss et al., 2010). Third, whereas muscimol spread has been validated, to our knowledge, there is no study systematically comparing THIP with gadolinium or THIP/muscimol binding over a time course of 2–3 h, which is the typical session duration of task-related inactivation/fMRI studies. Thus, although the co-injection of gadoliunium or MnCl2 is exceedingly helpful to identify the center-of-mass of the pharmacological injections, it may over- or underestimate the total tissue volume that is affected.

### 3.3 Discussion and outlook

The general advantage of the reversible inactivation approach in NHPs is that baseline and inactivation sessions can be repeatedly interleaved, providing control for idiosyncratic behavioral and brain activity patterns. This is particularly relevant in cognitive tasks where initial biases, behavioral strategies and BOLD activation patterns vary between individuals, or exhibit longitudinal drifts. In addition, distal inactivation effects can be measured within minutes of the onset of drug action, and before long-term reorganization can take place. Local inactivation is also not expected to immediately cause changes in neurovascular coupling, as is seen in stroke patients (de Haan et al., 2013).

The studies reviewed in Sections 2 and 3 demonstrate the power of combining whole-brain imaging with localized permanent or reversible lesions to investigate the contribution of specific brain regions to a given cognitive function and to assess lesion effects on remote brain structures. Employing monkeys in this context is particularly important when brain structures of interest are rarely selectively damaged in humans e.g. due to vascularization patterns and when those brain regions are not found in rodents (e.g. medial pulvinar, superior colliculus, or specific subregions in the intraparietal or superior temporal sulcus). Thus, the insights derived from such causal perturbation work carry high translational value as they increase our understanding of the neurobiological mechanisms of normal function, the impact of brain damage in humans, and the resulting compensatory plasticity mechanisms that might potentially be harnessed to assist recovery. The beneficial involvement of the intact hemisphere after unilateral lesions in NHP, for instance, is supported by patient studies demonstrating recruitment of the opposite hemisphere during unilateral brain damage recovery (Bartolomeo and Thiebaut de Schotten, 2016; Umarova et al., 2016). Likewise, the importance of areas along the superior temporal sulcus for spatial orienting and attention, as demonstrated in NHP studies, is paralleled by an emphasis on the involvement of temporal and temporal-parietal damage in neglect patients (Karnath and Rorden, 2012). Subcortical mechanisms of spatial attention and decision making elucidated in NHP studies have also now been identified in neurological studies (Karnath et al., 2002; Weddell, 2004).

It is important to point out that the main translational value of the combined lesion-imaging studies in NHP might not be the direct development of therapeutic interventions but a better understanding of the neural network mechanisms underlying specific neurological symptoms, which is however required to inform therapeutic interventions. While the neuroimaging findings from the monkey studies can inform the analysis of fMRI data acquired in human patients, one needs to consider how experimentally induced cytotoxic or ablation lesions (Section 2) and transient reversible lesions compare with stroke or traumatic lesions in human patients, especially with respect to long-term reorganization and compensation mechanisms.

There are some important differences in etiology and time course between reversible inactivations in NHPs and stroke-induced lesions in human patients. For instance, the human stroke-induced lesions are typically more extensive, affecting also fibers of passage and neurovascular coupling in surrounding regions (de Haan et al., 2013; Johansen-Berg, 2007), while GABA-A agonist injections do not compromise those aspects. In addition, stroke may trigger inflammatory and compensatory molecular cascades that are not present with pharmacological inactivation (Mohajerani et al., 2011). Human patient fMRI measurements take place at the acute and chronic phases, weeks/months after the lesion, allowing for long-term reorganization to take place. Thus, permanent lesions (Section 2) are a better stroke model, while reversible inactivation studies provide greater specificity and clarity of insights on neuronal mechanisms and short-term effects. The neurobiological substrates of visuomotor and attentional functions that are commonly thought to be affected in neuropsychological conditions such as spatial neglect show strong hemispheric asymmetries and weaker contralateral tuning in humans than in monkeys (Kagan et al., 2010). Spatial deficits following lesions in the right hemisphere are more pronounced in humans compared to the typically more moderate and symmetrical effects in NHPs (Gaffan and Hornak, 1997 Mesulam, 1999; Oleksiak et al., 2011). Despite differences between species, NHP findings often con-verge with results from the human clinical literature. This underscores the translational potential of the approach, which is now well-positioned to guide the selection of target regions for therapeutic interventions in humans such as stimulation of functionally identified compensatory circuits (Wilke et al., 2014). To further advance insights into network dynamics and compensation, an exciting path forward lies in manipulating several brain regions, including by combining reversible lesions with ‘facilitatory’ perturbations such as electrical, ultrasound or optical stimulation.

## 4 PET neuroimaging with brain perturbations

### 4.1 FDG-based approaches

Because of its physiological kinetics, the combination of 18-fludeoxyglucose (FDG) and Positron Emission Tomography (PET) scanning provides a useful tool to examine metabolic changes in freely behaving animals. FDG is a radiolabeled glucose analog that gets trapped in metabolically active cells, where it remains until the fluorine-18 decays. Because the half-life of fluorine-18 is ~100 min, animals can be injected with FDG, freely behave for 30–45 min during FDG uptake, and then be anesthetized for PET scanning to examine regional metabolism that is concurrent with behavior. This approach is ideal to demonstrate context-specific effects of regional manipulations on distributed brain function in freely behaving animals. Injection routes for the ligand can vary. In marmosets, a vascular access port is commonly implanted subcutaneously in a single surgery a week or so before scanning. This allows the ligand on the day of the scan to be administered subcutaneously but with direct access to the jugular vein ensuring rapid delivery to the brain. In macaques, FDG can be injected through an intravenous (IV) catheter in the saphenous vein.

#### 4.1.1 FDG and lesions

FDG-PET has been used to assess the effects of localised cytotoxic lesions and ablations in the prefrontal cortex on downstream circuits involved in threat processing. Human neuroimaging studies had identified inverse associations between the activity of the prefrontal cortex and the amygdala in relation to the regulation of negative emotion (Ochsner et al., 2004) but the causal nature of these associations had not previously been determined. To address this issue, a unilateral lesion model was adopted to assess the effects of localised lesions of either the left or right anterior orbitofrontal cortex or ventrolateral prefrontal cortex within individual marmosets by comparing the lesioned side with the intact control side. This approach is possible because the projections between the prefrontal cortex and its downstream subcortical targets are primarily unilateral (Roberts et al., 2007). Marmosets were exposed to two sessions of threat, before and after a session of safety. In each session, they received administration of FDG and after 30 min of threat or safety exposure, FDG uptake on the intact and lesioned sides was measured under anaesthesia. Bilateral cytotoxic lesions (specifically targeting cell bodies and leaving intact fibres of passage) of either prefrontal region heighten anxiety-like behavior in response to distal threat in the form of a human intruder (Agustín-Pavón et al., 2012). In the unilateral FDG study, FDG uptake in the dorsal amygdala and the neighboring anterior insular cortex was higher during threat compared to safety on the control side, illustrating that activity in both these regions was differentially regulated by the level of threat in the environment. In contrast, this differential uptake between threat and safety was not seen on the lesioned side. Instead, FDG uptake was as high during safety as during threat, revealing that the threat generalisation effects of lesions in two separate prefrontal regions converged on the amygdala and insula (Shiba et al., 2017).

In macaques, Fox & Kalin have extensively used FDG-PET to identify the distributed circuit that underlies individual differences in anxietylike responses in > 600 animals, by injecting FDG before exposure to a potentially-threatening human intruder making no-eye contact (NEC) (Fox et al., 2015a, 2008, 2005; Kalin et al., 2005; Oler et al., 2010; Shackman et al., 2013). To investigate the contribution of prefrontal cortex, Fox and colleagues used FDG-PET to demonstrate that orbitofrontal cortex (OFC) ablations were sufficient to decrease metabolism in the bed nucleus of the stria terminalis (Fox et al., 2010), a core component of the neural substrate that underlies dispositional anxiety (Fox et al., 2015b; Fox and Shackman, 2019). This approach provides a unique look into the causal effects of one brain region on context-specific metabolism in other regions.

#### 4.1.2 FDG and temporary manipulations

FDG has also proved effective in identifying changes in network activity associated with temporary manipulations. Subcallosal cingulate cortex has been implicated in depression and other stress related disorders. PET imaging has revealed increased activity associated with the severity of the disorder itself (Fitzgerald et al., 2008), whilst activity reductions have been correlated with successful treatment following a range of therapies including pharmacological (e.g. fluoxetine) and surgical (e.g, deep brain stimulation) (Mayberg et al., 2005). To determine causality in relation to heightened activity in this region and symptoms such as anxiety, anhedonia and cardiovascular dysfunction, temporary overactivation of this region was induced in marmosets using the glutamate transporter (GLT1) blocker, dihydrokainic acid (DHK). The resulting overactivation reduced anticipatory cardiovascular and behavioral arousal to an appetitive Pavlovian conditioned cue as well as the maximum number of responses an animal was prepared to make to obtain reward using a progressive ratio task. These results were accompanied by heightened reactivity to distal and proximal threat as well as basal cardiovascular dysregulation, which are all symptoms associated with stress-related disorders. FDG-PET, comparing saline controls and DHK infusions not only confirmed a DHK-induced increase in FDG uptake in area 25 but also revealed a network of differentially engaged structures that depended on the context the animal was placed in. In an appetitive context, area 25 overactivation engaged regions of the dorsomedial prefrontal and anterior cingulate cortex and regions of the mid insula cortex (Alexander et al., 2019). In contrast, in a threatening context, area 25 overactivation engaged a subcortical threat processing network that included the amygdala and hypothalamus and at the same time dampened activity in higher-order prefrontal areas including area 46, frontal pole and orbitofrontal cortex (Alexander et al., 2020). Moreover, acute treatment with ketamine, an antidepressant shown to be rapidly effective in some treatment-resistant patients with depression, not only ameliorated the area 25 overactivation-induced blunting of anticipatory appetitive cardiovascular and behavioral arousal but also reversed the associated changes in brain network activity identified with FDG-PET.

#### 4.1.3 FDG and gene therapy

Gene manipulations can be combined with neuroimaging to assess distributed alterations in brain function that result from regional manipulations of a specific transcript. Like lesions, these techniques are often permanent or long-lasting, and allow for the examination of long-term consequences of brain manipulations on behavior, brain function, and brain structure. Unlike lesions, however, viral-gene manipulation can increase or decrease expression with molecular specificity. AAV-mediated gene up-regulation (adeno-associated virus), for instance, has been used to upregulate corticotropin releasing hormone (CRH, AAV2-CMV-CRH) in the central nucleus of the amygdala, revealing distributed changes in brain function and structure, using FDG-PET, fMRI, and diffusion-weighted MRI (Kalin et al., 2016). These studies can complement RNA-sequencing association studies to demonstrate a causal relationship between gene expression and brain function. Along these lines, Fox and colleagues (2019) performed RNA-sequencing of dorsal amygdala tissue, which suggested that specific neuroplasticity-related processes, involving neurotrophic factor-3 (NTF3), may play a role in decreasing anxiety-like behavior. To test the causality of this association and its neural substrate, they injected an AAV5 expressing NTF3 (AAV5-CMV-NTF3) into the dorsal amygdala and found decreases in anxiety-like behavior with associated changes in metabolism throughout the brain’s distributed anxiety-related circuitry, including the bed nucleus of the stria terminalis (Fox et al., 2019).

Continuously evolving viral vector genetic technologies provide a unique opportunity to manipulate specific projections and/or populations of brain cells. Researchers are developing viral vector technologies to optimize delivery across the blood brain barrier (Flytzanis et al., 2020), and to specifically infect specific populations of cells. For example, Dimidschstein et al. (2016) developed a GABAergic-specific infection strategy by inserting the GABA-neuron-specific mDlx enhancer in an AAV. This approach was successful in infecting forebrain GABA-expressing neurons in marmosets. Similarly, using a dual infection strategy (Stauffer et al., 2016), injected the VTA of rhesus monkeys with a mixture of the Cre-dependent ChR2 optogenetic activator (pAAV5-DIO-Ef1*α*-ChR2(h134)-EYFP) and a virus expressing Cre attached to a tyrosine-hydroxylase (TH) promoter (AAV2/9-rTH-PI-Cre-SV40). This dual-infection strategy allowed for optogenetic control of the VTA neurons that expressed TH (i.e., dopamine neurons). Retrograde-transported viruses can furthermore be used to manipulate specific projections, for example to manipulate all neurons that project to a specific region. Combinations with an intersectional strategy are also possible, for instance by infecting one region with retrograde transported Cre, and a projecting region with a Cre-dependent viral vector. Together, these viral vector technologies are paving the way for sophisticated gene-up-regulation, optogenetic, and DREADDs studies.

There are various limitations of viral vector gene manipulations that are shared with DREADDs and optogenetic approaches, methods that also rely on viral infection. All these techniques rely on accurate targeting and infusions, up-take of virus into cells, and expression of the virus itself. Herpes simplex virus (HSV) remains the most effective strategy for infecting brain cells, but it is often suboptimal because it edits DNA and is considered harmful to cells. In many cases, AAVs are preferred. They are considered much safer and some have even been approved for use in humans. However, AAVs rely on cell-surface molecules to enter cells, and the distribution of these AAV-responsive elements does not seem to be uniform across the brain or conserved across species. Thus, identifying the appropriate viral vector for the region, species, and study remains critical.

### 4.2 PET with other ligands

In addition to FDG, PET can also be used in combination with other ligands to examine specific molecular pathways, such as dopamine, serotonin, and many others^1^. PET detects the decay of radioactive isotopes that are used to label ligands, so the ideal tracer depends both on an ability to conjugate the radioactive isotope to the molecule of interest, and the half-life of the isotope that determines the time course of the experiment. Common radio-active tracers for neuroimaging include Carbon-11 (^11^C), which has a half-life of ~20 min, Fluorine-18 (^18^F), which has a half-life of ~110 min, and Iodine-123 (^123^I), which has a half-life of ~13 hours. Isotopes with a short half-life, such as ^11^C, have to be made on-site, while others can be shipped from manufacturers. PET studies can track individual differences in binding potential and have been used to track serotonin transporter binding using ^11^C-DASB (Christian et al., 2009a; Golub et al., 2019), serotonin 1a and 2a binding with ^18^F-mefway (Christian et al., 2013) and ^18^F-altanserin (Santangelo et al., 2019) respectively, ^11^C-AZ10419369 to the 5-HT1B receptor (Yamanaka et al., 2014; Yang et al., 2019) and binding of the dopamine DRD2/3 receptor using 18F-Fallypride (Christian et al., 2009b; Clarke et al., 2014). Such studies have provided insight, for example, into alterations in serotonin signalling related to the serotonin transporter polymorphism in the upstream regulatory region associated with a trait anxious phenotype in macaques (Christian et al., 2009a) and marmosets (Santangelo et al., 2019), comparative binding of vortioxetine, a mixed antidepressant, and citalopram, a selective serotonin reuptake inhibitor (Yang et al., 2019), and ketamine (Yamanaka et al., 2014) to 5-HT1B receptors in macaques as well as striatal dopamine function following prefrontal manipulations in marmosets (Clarke et al., 2014).

### 4.3 PET combined with DREADDs

PET has also become an important component of the chemogenetic technologies for circuit analyses. Recent advances in chemogenetic technologies allow for bidirectional control of neurons using viral vector-mediated expression of designer receptors exclusively activated by designer drugs (DREADDs). As the name suggests, DREADDs are receptors that are not naturally occuring in the brain, that bind drugs that are also not naturally present. The typical DREADDs study involves surgery to express the receptor, by infecting cells with a viral vector to increase expression of the receptor, then delivery of a drug that binds these receptors. DREADDs are typically G-protein coupled receptors and can be excitatory or inhibitory (i.e., Gq, Gi, and Gs). DREADDs allow for reversible activation or inhibition depending on what DREADD is expressed. The most common implementation of DREADDs were derived from muscarinic receptors and designed to excite (with hM3Dq) or inhibit (with hM4Di) neurons by binding the designer drug Clozapine-N-Oxide (CNO), but others tools are also available. DREADDs are used in primates to activate or inhibit OFC (Eldridge et al., 2016), amygdala (Grayson et al., 2016; Raper et al., 2019), dlPFC (Upright et al., 2018), to name a few. Because the DREADDs approach does not require implantation of stimulation equipment, it is highly compatible with neuroimaging. One study, in particular, infused AAV-hSyn-hM4D-mCherry to inhibit amygdala neurons in anesthetized (1.3%-1.7% isoflurane) rhesus monkeys undergoing fMRI (Grayson et al., 2016). This study found disruptions in amygdalo-cortical and cortico-cortical functional connectivity as a result of DREADDs activation. However, the global network structure is better preserved following DREADDS inhibition of the amygdala than after permanent cytotoxic lesions to the hippocampus (compare Fig. 6 in Grayson et al., 2016 with Fig. 6 in Froudist-Walsh et al., 2018a). This suggests that either the hippocampus has a greater role in facilitating brain-wide connectivity or, more likely, that plastic reorganisation of connections is much more extensive in the weeks and months following permanent lesions than in the minutes and hours following DREADDS suppression.

DREADDs have a number of advantages, including the ability to manipulate large areas and/or specific populations and projections. DREADDs are limited by the viral-technique used to infect cells, and region- and species-specific infection should be verified. In addition, a number of groups have raised concerns about the application of DREADDs technologies that are important for consideration in the primate world. In rhesus monkeys, DREADDs with an mCherry conjugate tend not to be expressed on the cell-surface, preventing CNO binding (Galvan et al., 2019). This can be alleviated by using an HA-tag instead of an mCherry tag.

DREADDs can also be limited by the use of CNO, particularly at the large doses required for use in NHPs, which sub-optimally crosses the blood-brain barrier and can be back-metabolized into Clozapine (Gomez et al., 2017), a drug which itself can bind numerous endogenous receptors. A potential work-around for this problem is to use related ligands that bind the HM3Dq and HM4Di receptors including deschloroclozapine (DCZ), perlapine, Compound-21 (C21), or other designer ligands (Bonaventura et al., 2019; Chen et al., 2015; Nagai et al., 2020). Compared to CNO, a number of these ligands, including DCZ, have better blood-brain penetrance, similar specificity, and are less likely to be back-metabolized into other psychoactive molecules. A related concern for each of these ligands is the potential for off-target effects, as the proposed designer drugs share some affinity at other receptors. Importantly, this can result in off-target effects that are behaviorally relevant and potentially variable between individuals (Upright and Baxter, 2020). To address this concern, Upright and Baxter (2020) stress the importance of within-subject controls in target tasks, to ensure observed effects are not due to non-specific binding at other receptors.

Because DREADDs are cell-surface receptors, PET imaging can be used to localize DREADDs by radio-labeling ligands that bind to DREADD receptors. This has been done using well-established (i.e. ^11^C-Clozapine), as well as novel (e.g. ^18^F-JHU37107, and ^11^C-DCZ) ligands (Bonaventura et al., 2019; Gomez et al., 2017; Nagai et al., 2020, 2016). This approach provides a unique in-vivo demonstration that DREADDs are effectively expressed, without performing a terminal experiment, making it ideal for use in non-human primates.

DREADDs can be used to modulate activity in specific cell populations across a large area, making them well suited to provide both microscopic and mesoscopic insights. DREADDs are ideal for manipulating large areas, as might be required to alter striatal function in diseases like Parkinsons, or any other disorder that results from dysregulation of distributed circuitry. Numerous challenges remain to be optimized before NHP DREADDs can be a commonplace neuroscientific tool, but recent advances in viral-vector technology, genetically encoded receptors, and designer ligands, combined with a practical experience make DREADDs an extremely promising technology for use in NHPs. DREADDs are limited by the time course of drug update and binding, but are extremely flexible in how they can be used. There is no in-dwelling equipment, simplifying DREADDs-fMRI/PET experiments and enabling study of freely moving animals. Moreover, radio-labeled ligands can be used to ensure the efficacy of transfection in vivo. In short, DREADDs are an exciting addition to the toolkit for testing causality in NHP neuroscience.

### 4.4 Discussion and outlook

As with many other imaging technologies, PET can dissect out the effects of localised permanent or temporary manipulations on overall brain circuitry and neurochemistry in vivo. Consequently it has enormous potential in those studies establishing causality between symptoms of various psychiatric, neurodegenerative and neurodevelopmental disorders and the accompanying structural and functional changes in the brain. It can also be used to determine the effects on brain circuitry and neurochemistry of treatments used to ameliorate symptoms, whether these treatments are pharmacological, psychological or physical interventions, to help identify the circuits upon which these treatments act to have their efficacious actions. FDG-PET, in particular, has the advantage over other imaging modalities in identifying changes in activity in freely moving animals and the ability of PET to visualise other neurochemical systems makes it an extremely versatile tool for studying novel pharmacological compounds in collaboration with pharmaceutical companies and for testing the functional efficacy of chemogenetics.

## 5 Electrical stimulation, DBS and fMRI

### 5.1 Electrical stimulation and neuroimaging

The use of electrical stimulation in neuroscience has a rich history going back to studies of motor cortex function in the 19th century (Ferrier, 1876; Fritsch and Hitzig, 1870; Taylor and Gross, 2016). It has been the driving force behind the functional mapping of the cerebral cortex in human patients undergoing surgical treatment (Penfield and Boldrey, 1937) and was instrumental in discoveries such as the brain’s reward processing system (Delgado, 1964; Heath, 1963 Olds, 1958). Therapeutic applications are found in the cortical stimulation of sensory cortices for brain-computer-interfaces (BCI’s) and deep brain stimulation (DBS) treatments for movement or psychiatric disorders (Roelfsema et al., 2018).

**Fig. 4:**
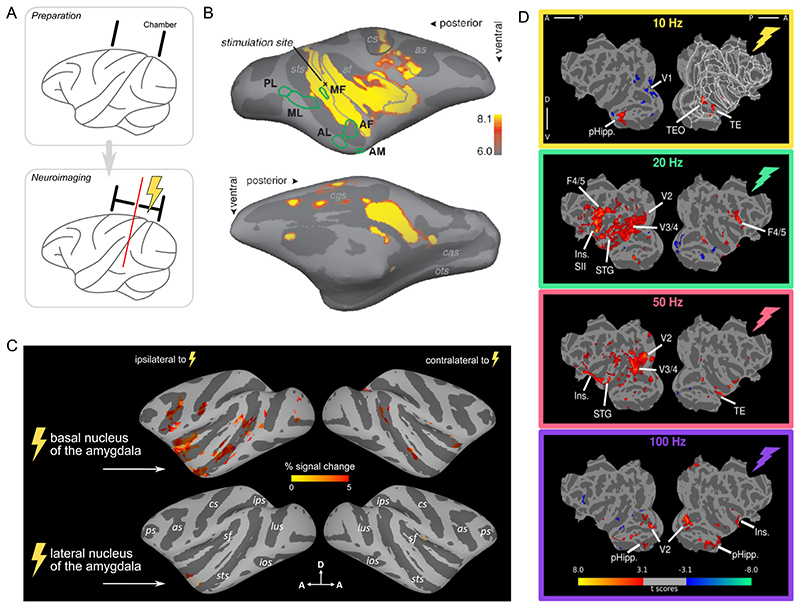
Electrical stimulation and neuroimaging. A) Schematic of the technique. To prepare for neuroimaging experiments, the animal is implanted with a chronic electrode or (as depicted) a recording/stimulation chamber for acute electrode penetrations. During imaging, electrical stimulation is delivered through an implanted electrode or one that is guided each session through a localization grid to the desired depth. B) Microstimulation-evoked activation patterns overlaid on the inflated right hemisphere (outlines of the face patches in green). The medial view reveals activation of cingulate cortex and adjacent somatosensory and supplementary motor areas. Adapted from Moeller et al. (2008). C) Significant activation maps arising from two stimulation sites in the left amygdala are shown on an inflated mid-cortical surface (uncorrected level, *p <* 0.001; cluster correction, 4). A site in the basal nucleus (top) activated the ipsilateral frontal, insular, temporal, and occipital cortex; all regions to which the basal nucleus is known to project. As many of these areas send no reciprocal projection to the basal nucleus (or to the amygdala at all), these activations most likely reflect orthodromic propagation from the amygdala. Several of these regions were also activated contralateral to the stimulation. Stimulation at a site in the lateral nucleus (bottom) generated activity largely confined to the ipsilateral temporal pole and rostral auditory areas; regions that are reciprocally connected to the lateral nucleus. There was no activation in several regions that provide nonreciprocal input to the lateral nucleus, suggesting that antidromic activation was weak or absent. D) Frequency-specific MRI responses evoked by VTA stimulation. T-score maps of the stimulation frequency versus baseline (uncorrected level, *p <* 0.001; cluster correction, 20) overlaid onto cortical flatmaps in the D99 template space. Regions showing significant activation are indicated on the maps. Adapted from Murris et al. (2020). (as: arcuate sulcus; cas: calcarine sulcus, cgs: cingulate sulcus, cs: central sulcus; ios: inferior occipital sulcus; ips: intraparietal sulcus; lus: lunate sulcus; ots: occipitotemporal sulcus, ps: principal sulcus; sf: Sylvan fissure; sts: superior temporal sulcus).

The combination of electrical stimulation and functional neuroimaging (es-fMRI; Fig. 4 A) in NHPs first appeared in the studies of Logothetis and colleagues focusing on occipital brain regions in anaesthetized macaques (Tolias et al., 2005), and soon after on the wholebrain neuroimaging of awake and fixating animals (Ekstrom et al., 2008; Moeller et al., 2008). The advent of es-fMRI in NHPs has increased our understanding of anatomical connections and functional dynamics within complex brain networks (Ekstrom et al., 2008; Logothetis et al., 2010; Moeller et al., 2008; Petkov et al., 2015). In human patients, the impact of electrical stimulation has been visualized with MRI (Rezai et al., 1999), safety issues have been extensively considered (Rezai et al., 2005, 2002), and are being addressed for clinical applications (Oya et al., 2017). In the following, we consider the immediate effects of stimulation, as well as long-term consequences beyond the immediate stimulation epochs typically addressed in DBS approaches.

The term ‘es-fMRI’ is used throughout this paper in the broad sense of combining electrical stimulation with fMRI. In experimental neuroscience with NHPs, the electrical stimulation is most commonly done with micro-electrodes that have a diameter in the order of micrometers and deliver very focal stimulation with relatively low currents (i.e., microstimulation). Studies in humans are more commonly performed with macro-electrodes for which the size of the contact elements is in the order of millimeters. The distinguishing feature of DBS is the targeting of deep brain regions, not necessarily the type of electrode. While clinical DBS in humans is always done with macro-electrodes, animal DBS studies can either be performed with macro-electrodes to mimic the clinical situation, or with micro-electrodes in order to stimulate with higher spatial precision. While these approaches may not necessarily yield the same results at the global systems (mesoscopic) or neuronal-network (microscopic) levels, even when stimulation patterns are matched, both are valuable tools to improve our understanding of the clinical mode of action of DBS and the functional organisation of the underlying neural substrates.

The comparison of brain activity during periods with and without electrical stimulation can reveal brain activity components that can be attributed to the stimulation. The BOLD and CBV changes measured with fMRI are thought to reflect the electrically-induced stimulation of neurons near the electrode, which elicits neuronal spiking in the stimulated area and postsynaptic activity in neurons in the same area and in projection targets of the locally activated neurons. These projections can be both local and distant, and both direct and indirect, as is discussed in more detail below.

A first application of es-fMRI is to probe ‘functional anatomical connections’ or ‘effective connectivity’ in a living animal, i.e. anatomical projections that transfer stimulation effects from the local stimulation site to a distal projection area. Such microstimulation-induced activations go beyond purely anatomical connections in that the stimulation evokes distal functional activity modulations that can interact with sensory stimulation (Ekstrom et al., 2009, 2008) and influence the behavior of the animal. The approach also goes beyond ‘functional connectivity’, a term commonly used to describe correlated activity dynamics across areas without necessarily taking the anatomical connectivity pattern into account (Matsui et al., 2011). The term ‘effective connectivity’ is often used to emphasize the causal and potentially directional nature of the evoked responses in the anatomically connected region, but this term is also used in a more abstract way in a modeling context that can distinguish directionality (Stephan and Friston, 2010). In what follows, we will use the term ‘effective connectivity’ in the narrow definition of microstimulation-induced activity modulations in areas not directly stimulated.

#### 5.1.1 Mapping neural circuits

The es-fMRI approach in NHPs has been useful in studying effective connectivity within sensory and cognitive systems. In the auditory system, electrical stimulation combined with fMRI has been used to chart fronto-temporal effective connectivity. The idea here is that es-fMRI can be used to stimulate a cortical region, assess the fMRI activity response in other areas (presumed targets of the region that was stimulated) and then apply es-fMRI to one of these activated regions to reveal further stages in the circuit (Fig. 4 B). Petkov et al. (2015) used this approach to chart effective connectivity within cortical areas and found that some auditory cortical regions activate prefrontal cortex, while temporal lobe sites that are further downstream in the processing hierarchy appear to take different processing pathways to the frontal cortex.

**Fig. 5:**
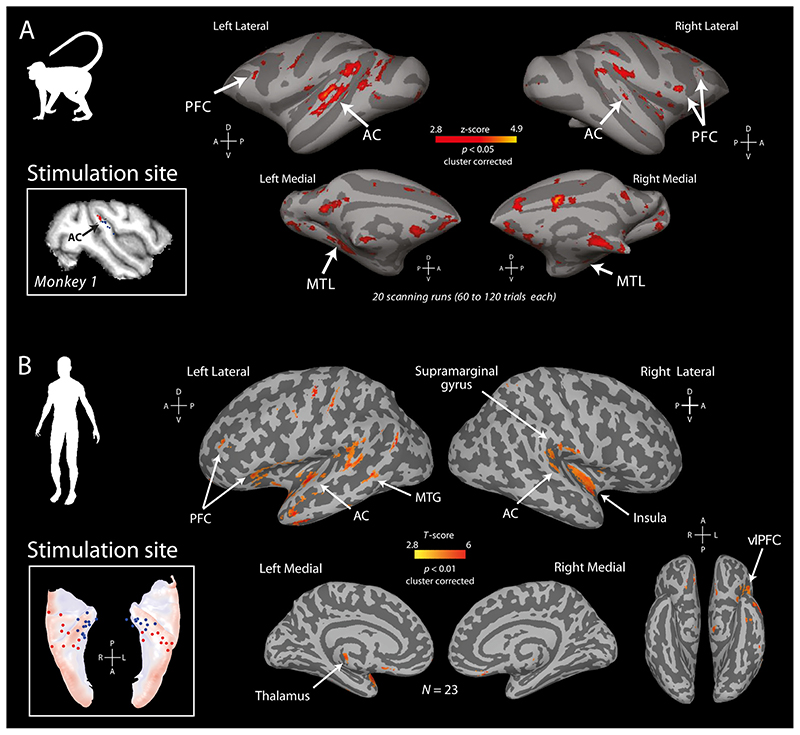
Comparison of es-fMRI in macaques and humans. A) Auditory cortex (AC) stimulation sites in one of the monkeys (inset). Es-fMRI group results from two animals show significantly activated voxels projected to the surface of a standard macaque template brain. B) Human es-fMRI of auditory cortex: Heschl’s gyrus on the superior aspects of the temporal lobe (inset). Human group results. Abbreviations: auditory cortex (AC), prefrontal cortex (PFC) and medial temporal lobe (MTL). Adapted from Rocchi et al. (2021).

Recently, es-fMRI has been directly compared between monkeys and humans. Rocchi et al. (2021) assessed fMRI results from stimulation of monkey and human auditory cortex (Fig. 5). They assessed the pattern of es-fMRI effects in frontal and medial temporal lobe regions evoked by stimulating different sites in the auditory cortex on the supratemporal plane. They observed remarkably similar patterns of activation in inferior frontal and parahippocampal regions in both species. Some of the monkey results also recapitulated auditory cortex to frontal cortex effective connectivity expected from neuronal tracing studies (Romanski et al., 1999). There is now also a human es-fMRI resource that has been established (Thompson et al., 2020), which is expected to grow as the neurosurgery community contributes additional human es-fMRI data from brain sites stimulated during fMRI for clinical purposes. Currently much of the data comes from stimulation of the amygdala and hippocampus, which are clinically assessed for epileptiform activity prior to a patient’s surgical resection.

#### 5.1.2 Interactions with sensory and cognitive processes

Although some studies have shown that es-fMRI effects do not interact with stimulus driven activity in the visual processing pathway (Moeller et al., 2008), a study targeting the frontal eye fields (FEF) in awake animals showed both positive and negative interactions between electrically- and visually-driven activity in the occipital cortex, recapitulating attentional modulations of visually-driven activity (Ekstrom et al., 2008). Stimulation of FEF had attention-like effects on the contrastresponse functions in visual areas (Ekstrom et al., 2009), adding to the evidence that FEF is causally involved in attention-driven modulations of sensory signals, a notion that was previously also shown at the single unit level in area V4 (Moore and Armstrong, 2003). Stimulation of FEF also causes task-dependent modulation of activity in the visual cortex (Premereur et al., 2013). Similarly, the stimulation of the dorsal pulvinar or area LIP during memory saccades or fixation enhances the activity in distal regions in relation to their original spatial selectivity in a task-dependent manner (Kagan et al., 2021). Finally, by combining electrical stimulation of deep brain neuromodulatory centers with fMRI, one can observe stimulation-induced cortical plasticity, and derive the role of neuromodulators in functional brain circuits (Arsenault et al., 2014, 2013; Arsenault and Vanduffel, 2019; Murris et al., 2021). Although structural connectivity is relatively fixed, synaptic connectivity changes dynamically and task related es-fMRI effects in behaving animals can thus be expected. Accordingly, the combination of es-fMRI with either sensory, cognitive or motor tasks provides an important tool to investigate the contribution of a targeted area in the underlying neural and behavioral processes.

#### 5.1.3 Deep brain stimulation (DBS)

Electrical DBS has emerged in recent decades as an important invasive treatment for a host of debilitating brain disorders, including Parkinson’s disease (Benabid, 2003 ; Benabid et al., 2009; Limousin et al., 1998), dystonia (Hu and Stead, 2014) and intractable psychiatric disorders such as obsessive-compulsive disorder (Denys et al., 2010; Greenberg et al., 2010; Nuttin et al., 1999), depression (Mayberg et al., 2005), epilepsy, and disorders of consciousness (Schiff et al., 2007). While NHP models have been instrumental in these developments (Benazzouz et al., 1993), the underlying brain mechanisms of DBS remain poorly understood and only a few clinical groups use functional imaging in combination with DBS. After clinical implantation of a DBS system, the main output measure is typically behavioral (e.g., observing that motor behavior is normalized).

Combining DBS with imaging methods such as fMRI offers a unique opportunity to visualise the impact of DBS on brain activity. While it may sometimes be possible to investigate the brain-wide effects of DBS with simultaneous stimulation and fMRI in human patients (Rezai et al., 1999), the stimulation parameter space (e.g., amplitude, frequency, pulse-width, charge-balance) is very large and care needs to be taken to establish safety (Oya et al., 2017; Rezai et al., 2005 2001). Systematic investigation of a range of relevant stimulation parameters (and their combination) often takes many experimental sessions, something that is simply not feasible in human patients. In addition, because DBS will not only affect local neurons but also passing fiber bundles, distal DBS effects will depend strongly on the precise electrode location relative to the individual brain anatomy (and any interaction of location with stimulation parameters). Once implanted, DBS electrodes in human patients cannot be easily moved, which means that a systematic investigation of the effect of stimulation location would have to exploit natural variability in electrode placements and require very large sample sizes. In animal models, it is possible to systematically vary stimulation parameters and stimulation locations in the same individual over many repeated sessions (Knight et al., 2013 Min et al., 2012; Murris et al., 2020). With NHPs, it is furthermore possible to perform these stimulation experiments in awake behaving animals to investigate how microstimulation affects behavior (Arsenault et al., 2014; Arsenault and Vanduffel, 2019; Murris et al., 2021). Stimulation parameters for es-fMRI and DBS are typically rather different and there is a pressing need for more DBS-fMRI work in NHPs to inform clinical work with human patients. DBS-fMRI in NHPs would create an avenue for an increased understanding of the functional mechanisms of DBS to guide the development of novel or improved treatment strategies in humans.

### 5.2 Mechanisms of es-fMRI

#### 5.2.1 Direct and indirect activation of neurons

Electrical stimulation can elicit behavioral and neurophysiological effects in some oculomotor and motor regions at low currents (< 60 μA), but in many areas, such effects require currents larger than 100 pA. With es-fMRI, reliable functional activation typically requires currents of at least 100-250 pA. A notable exception is the es-fMRI studies of Ekstrom et al. (2008, 2009), where frontal eye fields (FEF) sites were stimulated at half the current needed to reliably evoke saccades using the same electrode (~30 pA) and elicited considerable es-fMRI activity in projection brain regions. The volume of directly activated neurons relates to the delivered current: *r =* √I/K, where r is the radius of passive current spread, I is the current, and K is a constant of pyramidal cell excitability. The value of K depends on the tissue in question, varying between 300 and 27,000 *μ*m/mm^2^, and was estimated to be 675 *μ*m/mm^2^ in V1 using 200 *μ*s current pulses (Tehovnik et al., 2006; Tolias et al., 2005). For a 250 *μ*A current, the radius of passive current spread is in the 0.1-0.9 mm range, with a value of 0.6 mm for *K =* 675 *μ*m/mm^2^. Even at the upper end of this range, the volume of directly activated neuronal tissue is slightly less than the typical volume of an fMRI voxel. Nonetheless, es-fMRI effects are mesoscopic rather than microscopic and involve the direct activation of many thousands of neurons.

#### 5.2.2 Orthodromic vs. antidromic stimulation propagation

It is important to consider whether observed effects of es-fMRI reflect only orthodromic propagation (i.e., in the direction of action potentials from the neuronal soma to axonal boutons), or also antidromic propagation (i.e., in the opposite direction from neuronal action potentials). After all, electrical stimulation will use axons as cables and, once depolarization levels are reached, propagation will occur in both directions. Thus, it is possible that es-fMRI reflects both orthodromic and antidromic activation and cannot, on its own, distinguish the directionality of the effective connectivity it reveals. Antidromic propagation could additionally travel down axon collaterals and activate their targets. This could result in activation of distal areas that are not directly connected to the stimulation site, but that, along with the stimulation site, receive input from a third area along a branching axon.

There are a few ways to try to resolve the question of the direction of propagation in es-fMRI. One is to compare es-fMRI results with anterograde and retrograde tracer studies. However, most cortical regions are reciprocally connected, so a given es-fMRI activation could reflect either orthodromic or antidromic activation. Clarification has come from stimulation of subcortical structures, which more frequently have nonreciprocal connections. For example, es-fMRI of the amygdala has shown that activation either predominantly or exclusively drives orthodromic propagation (Messinger et al., 2015). Stimulation in the basal nucleus of the amygdala activates many distant sites that are known to be recipients of basal nucleus projections but that do not project back to the basal nucleus and, in some cases not to any other part of the amygdala either (Fig. 4 C). Conversely, stimulation of the lateral nucleus activates anterior temporal lobe regions, with which it has reciprocal projections, but does not activate several sensory areas from which it receives but does not send projections. Thus, there is evidence for activation of orthodromic targets and a lack of evidence for activation solely through antidromic propagation.

Feedback and feedforward projections arrive in different layers of the cortex, the pattern of which differs in early sensory and primary motor areas. Es-fMRI conducted at a spatial resolution sufficient to resolve the cortical layers could be used to assess if activations likely arise from antidromic or orthodromic effects (Self et al., 2017). Such laminar specificity can be achieved with higher field strengths (Goense et al., 2007; Logothetis et al., 2010) and perhaps with chronically implanted receive coils (Zhu and Vanduffel, 2019), but the combination of layerspecific fMRI with electrical stimulation has not yet been reported. In electrophysiological studies, the distinction between antidromic and orthodromic stimulation effects is sometimes made based on the shape and selectivity of the stimulation-evoked response, or the interaction with ongoing sensory processes (Klink et al., 2017). The rather low temporal resolution of fMRI makes it difficult to derive similar distinctions directly from the imaging signal, but a comparison of es-fMRI and electrophysiological signatures may allow inferences about the nature of es-fMRI effects (Rocchi et al., 2021).

#### 5.2.3 Monosynaptic vs. polysynaptic stimulation propagation

A related important topic is monosynaptic vs. polysynaptic propagation of orthodromic activations and/or subsequent deactivations (Logothetis et al., 2010; Sultan et al., 2011). Several lines of evidence support polysynaptic propagation, especially in the awake state. The primary (either cortical or subcortical) site of microstimulation may partially determine the extent of the dynamic nature of the connectivity. The stimulation of somatosensory cortex and FEF evokes activity in the contralateral cerebellum (Ekstrom et al., 2008; Matsui et al., 2012), and conversely, the stimulation of cerebellum activates extensive cerebello-cerebral polysynaptic pathways (Sultan et al., 2012). The activation of contralateral cortical regions after dorsal pulvinar stimulation as well as prefrontal activation after ventral pulvinar stimulation furthermore suggest polysynaptic thalamo-cortical transmission, since the corresponding monosynaptic projections have not been reported to exist (Baizer et al., 1993; Kagan et al., 2021). Likewise, the stimulation of LGN in combination with V1 inactivation revealed that the observed superior colliculus activation was due to thalamo-cortico-tectal signal propagation rather than a direct antidromic pathway (Murayama et al., 2011).

Non-homotopic contralateral activations after cortical stimulation also point towards polysynaptic transmission, although these could also arise from heterotopic transcallosal connections. Such connections are, however, mostly between regions that are adjacent on the rostral-caudal axis and otherwise tend to be sparse. Thus, strong heterotopic contralateral activations are likely due to a transcallosal connection followed by an additional synapse. Some evidence suggests that fMRI activity arising from polysynaptic routes, such as in subregions of the hippocampus following stimulation of the superior temporal lobe, are weaker than those from monosynaptic routes (Rocchi et al., 2021). Further evidence that es-fMRI can lead to polysynaptic activation comes from Moeller et al. (2008), who found that stimulation at a site in the upper STS produced activation across a huge swath of cortex extending from -5 to +20 mm (relative to the Frankfurt zero stereotaxic zero axis) within the upper bank and fundus of the STS, as well as activation in cingulate and somatomotor cortex (Fig. 4 B). In contrast, identical stimulation in the lower bank of the STS at the same AP coordinate produced discrete clusters of activation within the inferotemporal cortex. This difference suggests that different parts of the brain may be more or less likely to induce polysynaptic activation when stimulated. A possible hypothesis is that more diffusely connected areas may be more likely to induce polysynaptic activation, a notion that could be important for improved interpretation of es-fMRI results.

#### 5.2.4 Indirect activation of neurons

There is convincing es-fMRI evidence in support of activation of higher order projections (i.e., targets of the targets) (Ekstrom et al., 2008; Matsui et al., 2012). Upstream neurons with afferents to the directly stimulated neurons can furthermore be affected via antidromic or orthodromic multisynaptic propagation (Murayama et al., 2011; Rocchi et al., 2021). In addition, both cortico-cortical pathways and subcortical-cortical pathways, such as thalamo-cortical, tecto-cortical and cerebello-thalamo-cortical connections, may be identified using es-fMRI (Field et al., 2008; Logothetis et al., 2010; Matsui et al., 2012). The extent of the locally activated neuronal population, as well as the extent of affected distal regions, depend critically on stimulation parameters (e.g. amplitude, frequency and duration of stimulation). This dependency can be exploited by repeatedly probing the same location with different stimulation parameters within or across scanning sessions (Murayama et al., 2011; Murris et al., 2020; Tolias et al., 2005) to unravel the structure of extended functional networks.

#### 5.2.5 Awake vs. anesthetized state

Es-fMRI has been conducted with awake, lightly sedated, and anesthetized monkeys. Wherever (indirect) comparisons can be made, both comparable and distinct effects are observed across anaesthetised and awake states. For instance, stimulation of the auditory cortex evokes a highly similar pattern of frontal activation in anesthetized and awake animals (Petkov et al., 2015 Rocchi et al., 2021). This is however an indirect comparison, and a more careful comparison in the same animals under anaesthesia or awake states would be needed to establish the extent to which effects would differ across the states (Murris et al., 2020). In another study, stimulation of exactly the same targets within the parietal cortex of the same subjects revealed highly similar activation patterns during anesthetized and awake states (Premereur et al., 2015), with the only caveat that higher currents (~double) were required for similar es-fMRI effects during anesthesia. At the same time, the stimulation of the ventral tegmental area (VTA) showed divergent stimulation frequency-dependent patterns in the anesthetized and awake states (Murris et al., 2020) (Fig. 4 D). Furthermore, the presence of task- or stimulus-dependent effects in the awake behaving state (e.g., as shown for VTA and FEF stimulation) limits the comparability to the anaesthetized experiments due to the task-dependent effective connectivity seen with es-fMRI (Arsenault and Vanduffel, 2019; Premereur et al., 2013).

Whenever es-fMRI is performed in anesthetized animals, special care should be taken that the anesthesia protocol is robust and balanced. The pragmatics of awake and anesthetized animal scanning as well as the influence of anesthesia on the neuroimaging signal are discussed in detail elsewhere (Basso et al., 2021), but briefly and in specific relation to es-fMRI, we can note that if anesthesia is too deep there might be a partial or total block of neural activity induced in areas affected by the anaesthetic agent. However, anesthesia that is too light or too variable might lead to spurious fluctuations of excitability and activation. The type of agent and maintained level of anesthesia should furthermore be comparable across animals, conditions, and sessions for closer comparisons.

#### 5.2.6 Comparison with other techniques

**Fig. 6:**
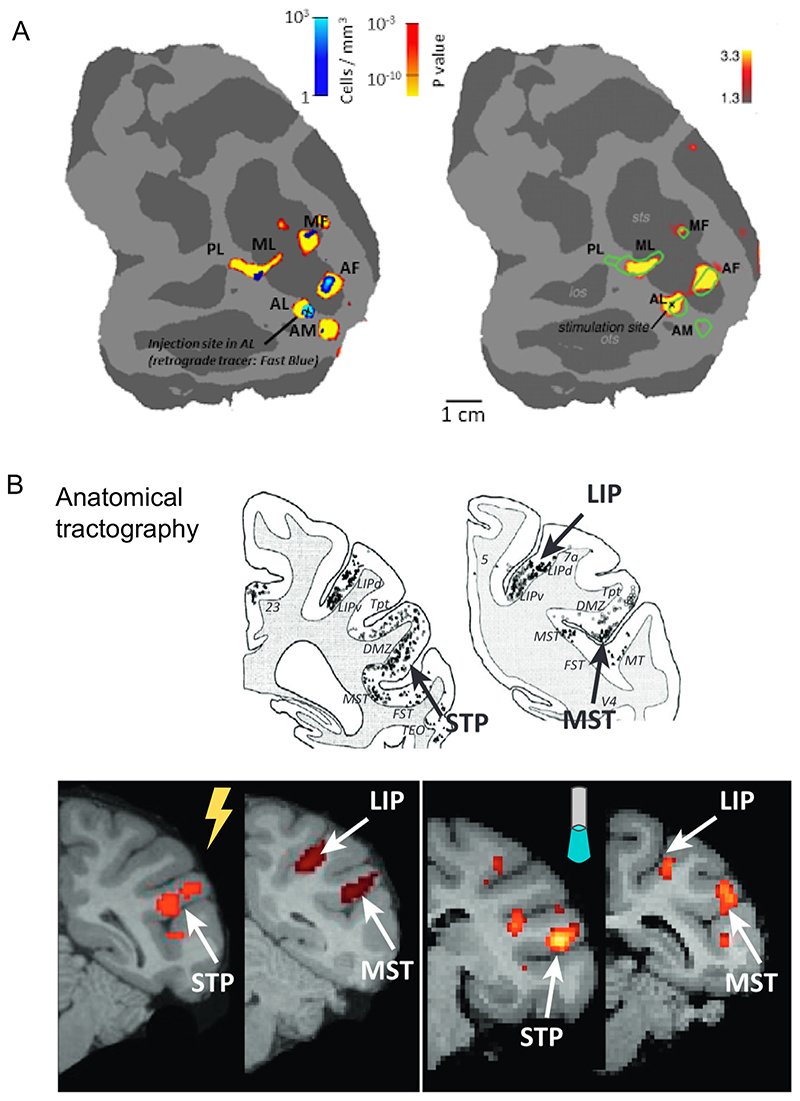
A comparison of brain-perturbation based connectivity and anatomical tractography. A) Es-fMRI effects and retrograde tracer injections in a macaque face patch within the same animal displayed on flat-maps of the right hemisphere. The left panel shows face patches (yellow) and density of labeled cells following injection of a retrograde tracer in face patch AL (blue). Note that clusters of remote, retrogradely labeled neurons were localized within four of the six face patches (ML, MF, AF, AM). The right panel shows brain regions activated by microstimulation of face patch AL. Remote activity was found in the same four face patches as revealed by tracer injection (ML, MF, AF, AM). Green outlines indicate face patches. Left panel: adapted from Grimaldi et al. (2016); right panel: adapted from Moeller et al. (2008). B) Injections of tracer in the macaque frontal eye fields (FEF) result in labeled cells in the lateral intraparietal area (LIP), the medial superior temporal area (MST) and the superior temporal polysensory area (STP) (Top; Schall et al., 1995). FEF microstimulation evokes fMRI activations in LIP, MST and STP (Ekstrom et al., 2008). Monkey optoMRI with ChR2-transduced neurons in FEF also evokes fMRI signals in LIP, MST and STP (Gerits et al., 2012). Adapted from Gerits and Vanduffel (2013).

Neuronal tracer injection studies are the gold standard for understanding microscopic anatomical connections. Despite the fact that es-fMRI lacks the precision of tracer studies, a number of studies have observed distal es-fMRI activations in areas that were previously identified as anterograde projection targets of the stimulated region using neuronal tracers (Petkov et al., 2015; Rocchi et al., 2021). Similarities between retrograde labelling and es-fMRI have been reported as well (Fig. 6). Discrepancies between es-fMRI and underlying ground truth anatomical connectivity have also been found. For example, Grimaldi et al. (2016) found very weak anatomical connections between inferotemporal and prefrontal face patches in a tracer study. By contrast, Moeller et al. (2008) found that es-fMRI in an inferotemporal face patch produced strong and specific activity in two prefrontal face patches. One important advantage of es-fMRI over tracer injections is that it allows one to use the obtained knowledge about connectivity from a specific animal to guide further investigations in the same animal, for instance with fMRI-guided electrophysiological recordings of single cell activity. Whereas, this is not possible with traditional tracer injections requiring post-mortem histological analysis. Using the approach of combined es-fMRI and fMRI-guided electrophysiology, Bao et al. (2020) discovered a new shape-selective network in macaque inferotemporal cortex specialized in representing spiky objects, a discovery that likely would not have been possible with post-mortem approaches precluding functional assessment of the identified network.

Es-fMRI can also be complemented and informed by multi-synaptic neuronal tracing methods or structural connectivity MRI data. Recent viral vector-based tracer approaches have been able to visualize hierarchical connectivity beyond the first synapse (Luo et al., 2008) and these approaches are now being extended to non-human primates (Xu et al., 2020a,b). The comparison of correlated activity patterns from resting state imaging (functional connectivity), structural connectivity patterns from in vivo imaging (e.g., DWI), and patterns of es-fMRI evoked activity can lead to important insights into the complex organisation that underlies the brain’s impressive processing capacity (Matsui et al., 2011; Murris et al., 2020).

#### 5.2.7 Negative fMRI signal stimulation effects

With a few notable exceptions, most es-fMRI NHP studies focus on positive BOLD or MION fMRI activations, i.e. the enhancement of the fMRI signal as a function of electrical stimulation. In the case of MION, where activity increases are characterized by reduced signal intensity, we take ‘positive activations’ to denote activations correlated with activity increases and thus with signal intensity decreases. Suppression of activity, or deactivation (relative to a no stimulation baseline), which is sometimes referred to as a negative BOLD, is also frequently observed. With higher-frequency stimulation (>50 Hz), negative es-fMRI effects are often less pronounced and consistent across runs or experimental subjects. The few available studies in NHP, mainly on subcortical structures, suggest that different stimulation frequencies may influence the preponderance of negative es-fMRI responses, as might the state of the animals and the properties of the stimulated cortical/subcortical region (Logothetis et al., 2010 Murayama et al., 2011; Murris et al., 2020; Sultan et al., 2011). Future work aiming to better understand the neural basis of negative es-fMRI effects is needed, and it would be useful to know if and how es-fMRI can reveal long-term effects of synaptic plasticity, such as frequency-dependent potentiation or depression (LTP/LTD).

### 5.3 Methodological considerations

#### 5.3.1 Practical considerations

Given the complex technical nature of es-fMRI, labs that are starting to use this approach might consider testing their complete set-up and procedures in sedated or anesthetized animals before studying awake animals. Most practical considerations outlined below apply to both anesthetized and awake subject experiments.

Electrical stimulation is delivered through a non-ferrous metal electrode, the active contact of which is placed in the desired brain location. The choice of stimulation electrode material matters as not all materials behave similarly when passing current. Most researchers use platinum or platinum-iridium electrodes due to their low corrosive properties and high charge transfer capacity (Brummer et al., 1983; Cogan, 2008; Tolias et al., 2005), but others have also used titanium electrodes due to their superior electrophysiological recording characteristics and stiffer material properties. Stiffness is important to allow straight penetration with minimal electrode diameter so that deep brain regions can be accurately targeted with minimal tissue damage (Bao et al., 2020 ; Moeller et al., 2008). The electrode can be chronically implanted, acutely positioned with an MRI-compatible microdrive, or lowered into place with a conventional microdrive that is then removed before entering the scanner environment.

Guide-tubes to aid electrode positioning can be made from an MR-compatible material such as fused silica or PEEK. They can be used to facilitate dura penetration and get the electrode to the target area in a straight trajectory. A second metal contact is typically placed in saline solution within the implanted chamber, or it is chronically implanted elsewhere to form a complete circuit. Connectors near the head of the animal should be void of nickel which is ferromagnetic and strongly affects the quality of the functional images^2^. The two wires attached to the contacts should be twisted together to prevent the formation of induction loops within the alternating magnetic field of the scanner that might induce currents. The wires are then typically fed into a shielded BNC cable and connected to a current isolator, which is controlled by a stimulator. The cable capacitance of this circuit should be minimized by choice of cable, with the cable being no longer than needed. Cable capacitance will impact the rise time of the stimulation current and might prevent the current delivered to the brain from ever reaching the specified level within the pulse (phase) duration. The output of the complete stimulation circuit should thus be measured, which can be done with an electrically isolated (e.g., battery-powered) oscilloscope. The resulting voltage waveform across a small series resistor can help assess the actually delivered vs. requested current.

A filter to shield the circuit from the Larmor frequency of the scanner should be added between the stimulus isolator and the electrode lead into the brain to minimize imaging artifacts and protect both the brain and the isolator from scanner-induced high frequency currents. The filter (e.g. a low-pass filter with a 50 MHz cutoff) should ideally be made of non-ferrous components designed to operate in a strong magnetic field and be placed in the scanner near the electrode. This will keep the effective length of the electrode “antenna” far from the wavelength of the inducing radiofrequency magnetic oscillations. The stimulation circuit must be isolated from ground and the receive coil circuits. To prevent malfunction, the current isolator and stimulator need to be in the control room, or alternatively well shielded and secured to a surface inside the scanner room. The strength of the applied current should be increased gradually to a desired amplitude in a new experimental animal or a stimulation site to ensure that the animal does not experience any observable adverse effects, and that no (unintended) eye or limb/body movements are evoked, as these will distort the functional imaging.

#### 5.3.2 Stimulation parameters

Most stimulators allow the experimenter to choose between constant voltage and constant current stimulation. The fact that the electrode impedance in the brain can vary over space and time makes voltage controlled stimulation imprecise in terms of reliable charge delivery, thus constant current stimulation with charge-balanced biphasic waveform is often preferred. Typically, the nominal current levels used in es-fMRI (100–250 μA and well above) are higher than in many behavioral studies, but this is not always the case. With stimulation of the pulvinar, for instance, reliable behavioral effects have been obtained with 150–250 μA (Dominguez-Vargas et al., 2017), whereas stimulation of the FEF with 30 pA already evoked saccades and reliable es-fMRI effects (Ekstrom et al., 2009, 2008). Stimulation parameters vary substantially across studies, but researchers typically use charge-balanced pulses and allow a brief pause even at higher-frequency stimulation for the required neuronal hyperpolarization in order for further action potentials to be generated. Most es-fMRI studies in NHPs stimulate the brain with several high-frequency (100 Hz and above) pulse trains to reliably elicit fMRI activity, either in blocked or in slow event-related designs. Single stimulation trains can however also have a clear impact (Arsenault and Vanduffel, 2019; Murris et al., 2021), offering exciting opportunities for identifying time-resolved dynamics and stimulus- or behavior-related contingencies during a specific task epoch. Elucidating frequency-specific stimulation effects is another research direction that will enhance the power of es-fMRI approach (Murris et al., 2020).

#### 5.3.3 Safety risks and solutions

During es-fMRI scanning, radio-frequency (RF) exciting pulses (MHz range) and magnetic gradient field switching (kHz range) can induce current in the intracranial contacts. The RF pulse can cause heating of the electrode contacts, which should be kept within the safety limit of less than 1 °C increase (Carmichael et al., 2012). Gradient-Echo EPI sequences that are commonly used for es-fMRI are low-SAR (specific absorption rate) sequences and do not usually cause heating above this limit. Gradient field switching may also cause induced current in the electrode contacts but at a much lower frequency, causing mainly peripheral nerve stimulation at charge densities that are well below the safety limit. The orientations of electrodes and leads in the scanner can have a large impact on the degree of heating. While the exact relationship between orientation and degree of heating can be complex, in general the electrode and lead orientations should be along the scanner’s Z-axis as much as possible and cable loops should be avoided. The choice of excitation coil also matters. For instance, standard human MRI’s body excitation coils tend to evoke larger SAR than (custom) local transmit coils, which can lead to stronger heating. Careful assessment of induced heating with computer simulations and actual measurements with electrodes taken in solution, artificial cerebrospinal fluid, egg albumin, or a phantom are highly recommended (Hawsawi et al., 2020). Electrode displacement due to the time-varying stimulation current within the static magnetic field is also a concern that needs to be tested as part of the safety assessment approach before involving experimental animals.

#### 5.3.4 Imaging artifacts

Even with careful consideration of the MRI-compatibility of used materials, there may still be distortions in the MRI signal induced by headposts, head caps, chambers or intracranial implants. Scanning sequences with short TE (< 30 ms) have less signal dropout. Spin-Echo EPI’s are less affected by artifacts than Gradient-Echo EPI’s but come with higher heating risk and lower BOLD sensitivity. RF artifacts and signal noise introduced by the presence of stimulation connections or active current delivery can be assessed with special quality assessment sequences. Alternatively, an adjusted standard functional EPI sequence with the transmit RF coil power set to zero can be used for artifact assessment. The latter option affords a convenient possibility to visualize any RF noise artifacts against the background level of noise in real-time, while connecting and adjusting the parts of the stimulation circuit and associated devices ideally one by one, with the exact same EPI sequence that will be used for actual functional data acquisition. For example, while the stimulation cable should be as short as possible, the exact placement and the path of the cable relative to the scanner bore can affect RF noise. Such RF noise debugging can first be done with a jelly or saline bath phantom approximating the head, and then on the experimental animal in each scanning session. Remaining scanning artifacts can be addressed during processing stages using fieldmap and other types of reference scans and analytical solutions (Tasserie et al., 2020).

### 5.4 Discussion and outlook

Electrical stimulation of the brain remains a major technological tool for neuroscience. As part of fundamental neuroscience, the controlled and focal electrical stimulation of a given brain area helps to better define and understand neural networks, circuits and their function. The link between neural activity and behavior can also be studied in a causal manner. In clinical neuroscience, deep brain stimulation (DBS) in NHPs led to major advances in the treatment of Parkinson’s disease (Mitchell et al., 2018; Roelfsema and Treue, 2014). DBS is further being developed to treat several debilitating neurological and psychiatric diseases such as obsessive compulsive disorder, severe depression and disorders of consciousness. However, the neural mechanisms of electrical stimulation/DBS and its global brain consequences in terms of neural circuits remain largely unknown and will continue to require nonhuman primate models for further translation of knowledge and technology to humans. These do not need to directly lead to human clinical trials to be useful. Functional neuroimaging offers a unique opportunity to visualize and better understand mechanisms and modes of action through es-fMRI. In fact, es-fMRI can measure the direct effects of electrical stimulation/DBS as well as the neuromodulation it induces on activation caused by stimuli or ongoing tasks. The es-fMRI activation maps would be the starting point for finer electrophysiological neural study involving the recording of neural ensembles with high temporal resolution, using es-fMRI guided neurophysiology. The approach can also benefit from focusing on stimulating or resolving effects at or on cortical layers or sets of layers to better understand neural circuits and feedforward/feedback pathways. Moreover, es-fMRI maps would make it possible to build computational models of electrical stimulation/DBS on global brain dynamics (Wang et al., 2015a, b).

## 6 Optogenetics fMRI

### 6.1 Targeting of specific neuronal circuits and populations

Optogenetics uses precise light-gated excitation or inhibition of neuronal spiking responses (Boyden et al., 2005; Deisseroth, 2015). This is done by injecting a viral vector (typically Adeno-Associated Virus, AAV or lentivirus) that allows neurons to express light-sensitive channels. When light of a specific wavelength illuminates the regions with the transfected neurons, these light-sensitive channels either open or close, thereby changing the membrane potential of neurons and modulating the generation of action potentials. Optogenetics in NHPs is an indispensable model for the development of various optogenetically based treatments for humans, such as visual restoration in blindness (Chaffiol et al., 2017; McGregor et al., 2020), or novel treatment options for movement disorders (Porras et al., 2012). Targeted optogenetics in NHPs also provides the opportunity to investigate how primate behavior and cognition relate to the activity of specific neuronal circuit elements (Tremblay et al., 2020).

Initial studies in NHPs highlighted the feasibility of interrogating cortical circuits with this technique (Diester et al., 2011; Han et al., 2011, 2009) and manipulating behavior (Cavanaugh et al., 2012; Gerits et al., 2012; Jazayeri et al., 2012; Ohayon et al., 2013). Over the past few years, important progress has been made in optogenetic targeting and electrophysiological assessment of specific cell types. Examples include the successful targeting and control of neurons in the koniocellular visual lateral geniculate nucleus pathway (Klein et al., 2016), Purkinje cells in the cerebellar vermis (El-Shamayleh et al., 2017), GABA-ergic interneurons in cortex (De et al., 2020; Dimidschstein et al., 2016), dopaminergic neurons of the midbrain (Stauffer et al., 2016), feedforward and feedback connections between motor thalamus and cortex (Galvan et al., 2016; Yazdan-Shahmorad et al., 2018b), transcortical connections between somatosensory and motor cortex (Yazdan-Shahmorad et al., 2018a), saccade-related projections from FEF to SC (Inoue et al., 2015), ocular dominance and orientation columns in V1 (Chernov et al., 2018), and feedback projections from V2 to V1 (Nurminen et al., 2018). Considerable progress has been made to assess the effect of optogenetic stimulation on cellular activity using electrophysiological methods, and even though optogenetically induced alterations of behavior in NHPs are still sparse, consistent progress is also being made in identifying the circuit-level mechanisms underlying sensation, cognition and motor behavior (reviewed in El-Shamayleh and Horwitz, 2019; Galvan et al., 2017).

### 6.2 Combining optogenetics with fMRI

**Fig. 7:**
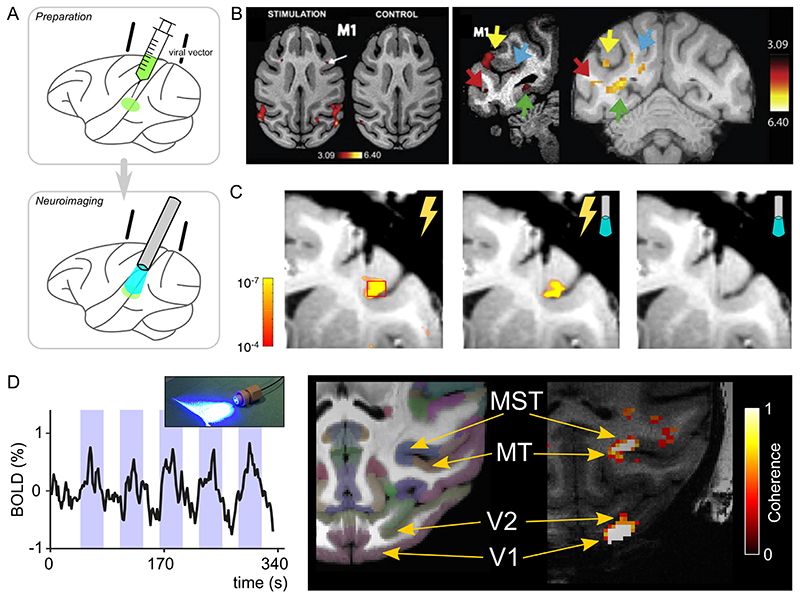
Optogenetic stimulation and neuroimaging. A) Schematic of the technique. Animals are typically implanted with a recording allowing the injection of a viral vector construct in a restricted part of the brain. In the neuroimaging experiment, areas that express the construct will be illuminated with light of a specific wavelength using either an optic fiber implanted into the brain or an LED light shining directly onto the brain surface. B) Activity induced by optical stimulation of FEF/F5 in the arcuate sulcus (T-score, *p <* 0.001, uncorrected). Control panels represent fMRI data after optogenetic stimulation of non-transduced sites nearby. Reproducible activations from different sessions were found in the visual cortex of monkey M1 (red arrow = area V4; green arrow = peripheral area V1; blue arrow = MSTv; yellow arrow = MSTd). Adapted from Gerits et al. (2012). C) fMRI activity close to the optrode tip during electrical (left), optical (right) and combined electrical and optical (middle) stimulation of FEF. Adapted from Ohayon et al. (2013). D) Coherence of BOLD activity (left) evoked by pulsed epidural optical stimulation of V1 (blue shaded areas) with a large-volume LED illuminator (left, inset) placed on top of the dura mater. The BOLD signal is highly coherent with the on/off switching of the LED light in V1 and visual areas to which it projects (Ortiz-Rios et al., 2018).

The combination of optogenetics and fMRI offers the opportunity to combine meso-scopic fMRI visualisation with neuronally specific stimulation targeting. At present, there are only two NHP studies that have assessed opto-fMRI effects (Gerits et al., 2012; Ohayon et al., 2013) (Fig. 7 A-C). Both studies targeted FEF as part of the primate prefrontal cortex involved in the control of eye movements and attention. The initial study by Gerits and colleagues used fMRI to guide injection of a pyramidal neuron targeting optogenetic construct (AAV5-CaG-ChR2-GFP) into the arcuate sulcus and FEF. They used Channelrhodopsin 2 (ChR2) which is a blue light-sensitive cation channel for neuronal depolarization. Optogenetic stimulation at 473 nm (40 Hz, 80 to 300 mW/mm^2^) using a laser-coupled optical fiber placed into the transfected region reduced saccade latencies by about 20 ms in a visuomotor task. fMRI measurements revealed activation directly at the FEF stimulation site and remotely in several areas in post-central gyrus and in visual cortex. These results complemented those from previous es-fMRI work (Ekstrom et al., 2008) and highlighted the extensive connectivity of FEF and its role in top-down modulation of sensory signals (Fig. 7 B).

The second fMRI study also relied on AAV5-based transfection into FEF and applied a promoter affecting mostly pyramidal neurons (Ohayon et al., 2013). Optogenetic experiments were combined with electrical microstimulation in order to directly compare the two methods. Though saccades could generally be evoked with less than 50 *μA* across electrical stimulation sites, optogenetic stimulation could only elicit saccades in a very confined part of the cortical tissue and when high frequency (80 Hz, 82 mW/mm^2^) stimulation was applied. However, when electrical and optogenetic stimulation were combined, optogenetic stimulation generally increased the probability of saccade occurrence. With fMRI measurements, electrical stimulation reliably drove fMRI activation in FEF and connected areas, but optogenetic stimulation alone appeared ineffective, as no functional activation in interconnected brain regions could be observed from ChR2 activation in either FEF or the other areas (Fig. 7 C). Control experiments using electrophysiology established that the optogenetic stimulation reliably drove neuronal spiking and histological analysis confirmed the presence of efficient construct expression in FEF. It is not clear how to explain the discrepant opto-fMRI findings between the two studies. Several methodological aspects might contribute, including the efficiency of neuronal optogenetic expression, which highlights the need for further study to optimize optical stimulation on fMRI in NHPs.

### 6.3 Neural transfection

Optogenetic manipulation largely depends on the efficacy and transfection of neuronal cell bodies with the viral construct (Mendoza et al., 2017). Previous NHP optogenetic studies highlight the need for large volume transfections of neural tissue as a step towards improving behavioral effects (Deng et al., 2017; Galvan et al., 2017; Han, 2012; Macknik et al., 2019). Larger volume transfections are possible by selecting viral vector serotypes that facilitate diffusion through brain tissue with some serotypes being more effective than others (Dodiya et al., 2010; Watakabe et al., 2015). Additionally, the density and volume of the transfected brain tissue will also depend on the neurosurgical approach used to inject the viral vector. Critically important is that viral vector constructs that are highly efficient in rodents, do not necessarily translate to primates. Hence, one either has to use constructs that already proved their efficacy in previous NHP experiments (Gerits et al., 2015; Scheyltjens et al., 2015 Watakabe et al., 2015), or one has to perform pilot tests to show its transduction efficiency. Methods used for viral vector delivery into brain tissue vary across NHP labs, with most groups relying on the use of standard microdiffusion based injections while others pioneered the utilization of convection enhanced delivery (CED) methods in NHPs where a catheter is placed in the area of interest for more efficient viral vector delivery (Khateeb et al., 2019 Yazdan-Shahmorad et al., 2018b). The latter approach was originally established for the treatment of tumours in humans using gene therapy techniques (Kells et al., 2009 ; Krauze et al., 2005). CED relies on bulk pressure and diffusion, decreasing the surgical time needed for injections (e.g. high infusion rates up 5000 nL/min) and results in a 10-100 fold increase in volume transfection as compared to standard injections (Kells et al., 2009). For further development of CED techniques, it is practical to first develop an MRI phantom for infusion testing prior to MRI-guided neurosurgery (Chen et al., 2004) and to consider the use of a step reflux cannula to avoid the solution diffusing upwards (Krauze et al., 2005). Some groups have also developed procedures for mass injections using multi-channel injection devices (Fredericks et al., 2020).

The use of MRI-based techniques for structural MRI provides invaluable information for neurosurgical planning, visualization and postoperative monitoring of the initial viral injections (see Box 1). Beyond standard T1-weighted imaging, T2 and diffusion-based images allow for the post-surgical monitoring of NHPs after injections by detecting increases in T2 signalling as a result of the fluid build-up in the tissue and by estimating the lack of diffusion within the targeted region. Supplementing the guidance of viral vectors using anatomical MRI (e.g. T1-weighted imaging) information, the use of task-based fMRI activations can confirm and greatly enhance the success of an opto-fMRI study (Gerits et al., 2016, 2012). Namely, a combined opto-fMRI-behavioral study, strong behavioral effects were observed only when a relatively small region was targeted with the results from the task-driven fMRI within the substantially much larger anatomical area, LIP (Gerits et al., 2016).

### 6.4 Light delivery to the brain

Another essential aspect of opto-fMRI studies in primates relates to the delivery of light into the brain. Opto-fMRI experiments require placing the optical source near the targeted brain region for depolarization of the transfected neurons (Lee et al., 2010). Given the larger brain size of primates (compared to rodents), optogenetic NHP experiments require an increase in the effective size of the illuminated brain tissue which often requires an increase in the size or power of the optical source. Neurons within the superficial cortex have been successfully targeted with a minimally invasive large-diameter MR-compatible high-power light-emitting diode (LED) fiber (1.5 mm diameter)^3^ placed on top of the dura mater (Ortiz-Rios et al., 2018) (Fig. 7 D). Combined with a step-function opsin with ultra-fast light sensitivity (SOUL), this minimally invasive approach facilitates light delivery from the brain surface (Gong et al., 2020). Another straightforward manner to increase the volume of brain tissue that can be optogenetically affected, is by using red-shifted opsins and red wavelengths that penetrate further in brain tissue (Chuong et al., 2014; Klapoetke et al., 2014).

An additional challenge in NHP optogenetics relates to the thickness of the dura mater which impedes light delivery into the underlying brain tissue. Practically, opto-fMRI experiments with minimally invasive surface stimulation likely benefit from a dura scrape prior to fMRI experiments which facilitates the penetration of light into the underlying neural tissue. However, detecting fluorescence with an intact dura in NHPs still remains a challenge with this approach. The insertion of an artificial dura can permit the penetration and absorption of light into the underlying transfected tissue (Ruiz et al., 2013). Importantly, the artificial dura allows in-vivo confirmation of fluorescence through the transparency of the silicon material, which provides visual confirmation of viral expression. The artificial dura combined with corticographical contacts and integrated optical sources dispersed through the film illuminates larger areas of cortical surface and reduces the effective power needed at any given source (Qazi et al., 2018). Most of these recent developments have been made in electrophysiological NHP studies with microelectrode recordings (Qazi et al., 2018) and calcium imaging experiments (Seidemann et al., 2016). The developed solutions remain to be implemented for opto-fMRI studies where the MRI-compatibility of the implant material and design will pose an additional technical challenge (Xu et al., 2019). An important aspect to consider regarding the artificial dura for functional MRI studies is the potential distortions that can be caused by the abrupt changes in magnetic field susceptibility around the material transitions zones (e.g. brain/artificial dura/bone), which can result in significant signal dropout. Moreover, long-term use of an artificial dura might result in thinning of the gray matter and increase the probability of a systemic infection if the artificial dura is not treated properly (Ruiz et al., 2013).

While cortical surface illumination could essentially be accomplished by epidural stimulation or through an artificial dura using minimally invasive fiber optical devices, deep brain stimulation requires the insertion of optical probes, which in the long term can affect tissue integrity and the disruption of white matter fiber tracts. The impact on brain tissue can be minimized with the use of tapered-end optical fibers (Acker et al., 2016, 2017) or by using multielectrode probes with integrated light source technology (NeuroLight optoelectrode, Plexon), which are unfortunately not yet available in an MR-compatible version. For the in-vivo detection of fluorescence, a feedback wavelength sensor coupled with the optic fiber allows differential light detection for the confirmation of functional transfections in deep brain structures (Diester et al., 2011).

### 6.5 Heat-induced confounds

An additional factor to consider for opto-fMRI studies relates to potential brain tissue damage due to the biophysical changes in temperature as a result of high-intensity light stimulation (Senova et al., 2017; Stujenske et al., 2015). Temperature changes can evoke spiking (Owen et al., 2019) and hemodynamic activity (Albers et al., 2019; Christie et al., 2013) even in the absence of opsin expression. This introduces a thermal confound in optogenetic studies that requires additional control experiments (i.e., similar stimulation of non-transfected brain tissue) (Stujenske et al., 2015). The optical irradiance required to drive light-sensitive channels within brain tissue is thought to be between 1 and 5 mW/mm2 for most NHP-available opsins (Klapoetke et al., 2014; Zhang et al., 2006). However, for targeting regions at much more distal targets (> 3 mm) much higher power, in the range of 200–500 mW/mm^2^, is theoretically required to effectively drive an opsin. Moreover, linear increases in power, duration and duty cycle can also result in a linear increase in temperature of neuronal tissue (Senova et al., 2017). To minimize temperature effects, it is recommended to use pulsed rather than continuous optical stimulation, particularly for opto-fMRI experiments where longer stimulation periods are required for blockdesign paradigms. In rodent studies, photostimulation parameters set at 40 Hz and at 200 mW/mm^2^ resulted in a temperature change of 0.2 °C in neural tissue for both blue and red light (Senova et al., 2017) and tissue heating near the fiber tip has been shown to result in negative BOLD responses (Albers et al., 2019). The blue-light that is typically used for optogenetic stimulation has a relatively high absorption in brain tissue, which increases the heating risk. As an alternative, red-shifted opsins are activated by longer wavelength light with low tissue absorption and moderate scattering. This approach can partially address the technical challenge of triggering light sensitive opsins located in deep structures, enabling a potential translational optical window for NHP optogenetics.

Finally, tissue heating can also be monitored using MRI thermometry (Rieke and Pauly, 2008).

### 6.6 Discussion and outlook

Optogenetics has profoundly changed the way neuroscientists approach the study of neural circuits. Optogenetics in NHPs in particular has tremendous translational potential to support the development of optogenetically-based treatments for visual restoration in blindness (Chaffiol et al., 2017; McGregor et al., 2020) movement disorders (Porras et al., 2012). But many technical and conceptual challenges still need to be overcome for more regular use in humans, and as such NHP development and testing remains important, including how optogenetics is combined with a macroscopic neuroimaging method such as fMRI. Scientists wanting to apply the technique should be aware that the ‘causal’ activation of specific cell types and circuits is quickly followed by downstream activation of connected neurons. Thus, while the original trigger for downstream brain activation or behavioral effect might be at the level of the initial stimulation site, it is possible that the true causal mechanism involves critical intermediate steps. Optogenetic inactivation of a circuit element might be a way to resolve this, but in practice this remains technically even more challenging. Similarly, stimulating or inactivating axonal projections from one area into another is still not routinely done due to the difficulty of electrophysiologically targeting axonal projections. Here, the combination of optogenetic stimulation or inactivation with fMRI holds great promise due to its wide areal coverage. Finally, optogenetic targeting, including its combination with fMRI, will benefit from the availability of more efficient and more selective neuronal transduction approaches (Bedbrook et al., 2019; Blankvoort et al., 2020; Deverman et al., 2016).

## 7 Ultrasound stimulation

### 7.1 Focused ultrasound stimulation and neuroimaging

**Fig. 8:**
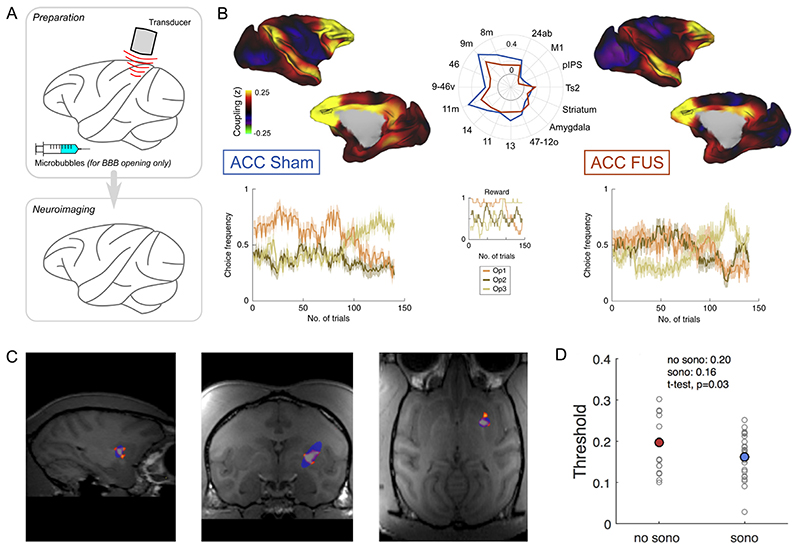
Ultrasound stimulation and neuroimaging. A) Schematic of the technique. Prior to neuroimaging, ultrasound stimulation is applied with an external transducer either with the systemic injection of microbubbles (for blood-brain-barrier opening) or without (for neuromodulation). B) Neural and behavioral results of FUS targeted at the Anterior Cingulate Cortex (ACC). The top row shows coupling of activity between the ACC and the rest of the brain for controls (Sham stimulation) on the left, and for ACC FUS on the right. The ‘connectivity fingerprint’ (middle) that can be extracted from these coupling patterns demonstrated clear effects of ACC FUS (blue: controls; red: ACC FUS). Adapted from Folloni et al. (2019). The bottom row shows behavioral effects of ACC FUS in a counterfactual decision task where animals chose between two presented stimuli, out of three possible stimuli (Op1-3; orange, dark green, light green) to obtain rewards. The reward probabilities associated with three stimuli varied over the timespan of an experimental session (middle). Without ACC FUS (left) decision frequencies for each option over time closely resemble the distribution of reward probabilities. ACC FUS disrupts this relationship suggesting a role for the ACC in translating internally tracked values into behavior. Adapted from Fouragnan et al. (2019). C) Contrast-enhanced (gadodiamide) MRI of blood-brain barrier (BBB) opening in the putamen using FUS with systemic microbubble injection. Blue oval indicates the planned target region. Red and orange voxels indicate actual BBB opening. D) Behavioral result of FUS sonication-induced BBB opening in the putamen. Thresholds (75% correct) from a coherent motion detection task are significantly lower after sonication. Small black dots are individual sessions, large colored dots are mean thresholds across sessions. C) and D) adapted from Downs et al. (2017).

Focused ultrasound (FUS) is a method for introducing mechanical energy into the brain using an ultrasound transducer (Fig. 8 A) operating at frequencies that are typically between 0.25 and 5 MHz. Early studies showed that FUS can alter the activity of electrically excitable cells (Harvey, 1929), suppress visually evoked potentials (Fry et al., 1958), and cause pupillary dilation when focused into the midbrain (Ballantine et al., 1960). One caveat associated with FUS in these pioneering studies was the need to perform a craniotomy prior to applying the ultrasound stimulation (sonication) (Fry et al., 1958). Recent technological advances (e.g., phased-array transducers) (Pernot et al., 2003) have enabled the fast development of low-intensity non-invasive transcranial FUS by allowing sonication to be conducted from positioning the transducer on the scalp, with sound waves passing through the skull and meninges and focusing to a region of interest within the brain (Elias et al., 2013).

Transcranial neuromodulatory studies of FUS were first conducted in lagomorphs and rodents (Kim et al., 2012; Tufail et al., 2010; Yoo et al., 2011; Younan et al., 2013a,b). Recent experiments in awake, behaving NHPs have shown neuromodulatory effects on oculomotor behavior (Deffieux et al., 2013; Wattiez et al., 2017) and decision-making (Bongioanni et al., 2021 Fouragnan et al., 2019; Khalighinejad et al., 2020; Kubanek et al., 2020). In combination with intravenous lipid microbubbles, FUS can also be used to increase the permeability of the blood-brain barrier (BBB), providing a method for targeted drug delivery of molecules that would otherwise be too big to cross the BBB (Hynynen et al., 2005, 2001; Tung et al., 2011).

Although FUS does not offer the same spatial resolution as optogenetic or chemogenetic approaches, it offers a key advantage of being a non-invasive brain stimulation technique (NIBS). In comparison with other NIBS, however, the main advantages of FUS are its better spatial resolution and the possibility of reaching brain targets that are over several centimeters in depth from the scalp. Finally, FUS is a nonionizing technique that is relatively simple and inexpensive to implement (Elias et al., 2013 Pouget et al., 2020; Tufail et al., 2011). A basic FUS experimental setup for NHP consists of a single element ceramic transducer. It can be combined with an integrated passive cavitation detector to monitor the cavitation signal. This can be made MR-compatible as needed. The transducer is driven by an RF amplifier (e.g., Electronics & Innovation A075, 75 W, 300 kHz -35 MHz). The input signal to the amplifier is produced by an arbitrary waveform generator (e.g., Keysight 32210A), which can be programmed via USB, or by a digital function generator (e.g., Handyscope HS5, TiePie engineering, Sneek, the Netherlands) controlled via Matlab routines. To fill the air gap between the face of the transducer and the subject’s scalp, the transducer can have a bladder-type coupling system that is filled with degassed water (e.g., Coupling Cone, Model C-101, Sonic Concepts). Degassed water may be replaced by or combined with polyvinyl alcohol (PVA) hydrogel (Lee et al., 2014). What matters is the use of an interface that will favor the undistorted propagation of the soundwave from the transducer to the skull. The depth of the ultrasound focus depends on the radius of the transducer, typically ~4 cm. The shape of the focus is dependent on the size of the transducer and its frequency (Holland et al., 2013). At the scalp, the area of the FUS beam is typically tens of cm^2^, reducing the risk of tissue heating or peripheral nerve stimulation. The FUS transducer should be calibrated with a hydrophone before use. A skull phantom is recommended to be inserted between the transducer and hydrophone to estimate pressure attenuation through the skull. Simulations may also be conducted for a better estimation of the pressure change at the focal point for each region targeted, e.g., using k-wave simulation (http://www.k-wave.org/). Positioning the transducer over the targeted brain region is achieved with a neuronavigation system (e.g., Brainsight, Rogue Research, Montreal, CA).

#### 7.1.1 Mechanism of FUS

The mechanisms through which FUS might affect neural activity are not fully known. Sound pressure can affect brain tissue physically through mechanical displacement, heating, and cavitation. It has been argued that the transient reversible effects of FUS that are currently of particular interest to cognitive neuroscientists are due to the mechanical effects of the propagating ultrasound wave that FUS generates affecting the neural tissue. Below 1 MHz or without the addition of intravenous lipid microbubbles, FUS effects are unlikely to be mediated by a cavitation mechanism. Temperature is also unlikely to be mediating neuro-modulatory effects if the acoustical energy is maintained to a low level (Constans et al., 2018), even though this mechanism is at the heart of high intensity FUS. At high intensity, FUS will create a non-reversible brain lesion, a thermal ablation phenomenon known since the earliest studies (Lynn and Putnam, 1944). Thermal ablation of focal brain regions is used clinically to treat patients suffering from Parkinson disease or essential tremor (Bretsztajn and Gedroyc, 2018; Elias et al., 2016).

Mechanical impact of soundwaves are thought to drive focused ultrasound neuromodulation (Blackmore et al., 2019; Kubanek et al., 2018; Menz et al., 2019; Tufail et al., 2011). The wide temporal range of FUS neuromodulation effects, with duration spanning from tens of milliseconds up to more than 90 min (Dallapiazza et al., 2018; Verhagen et al., 2019; Wattiez et al., 2017) suggests the involvement of different mechanisms. Fast-acting effects can be mediated by activating mechanically-sensitive ion channels (Kubanek et al., 2018, 2016; Tufail et al., 2011). Using acoustic radiation force imaging (ARFI), it has been shown that ultrasound waves can cause displacements from 1 to 3 microns (Bour et al., 2017; Ozenne et al., 2020; Phipps et al., 2019) in vivo in primate brains. Such displacement could alter membrane properties, which could also participate in the neuromodulation response to FUS (Blackmore et al., 2019). Nevertheless, this issue is debated (Yang et al., 2018), and other mechanisms such as sonoporation or the role of glia-neuron coupling in long-lasting neuromodulatory effects remain to be investigated.

While the aim of FUS is to perturb neural tissue, it is also possible that FUS could in some circumstances influence the vascular system (Verhagen et al., 2019), for instance, when the FUS focal point is close to a large vessel like the sagittal vein. Despite the fact that FUS uses sound frequencies well outside the range of normal hearing, there is evidence that FUS pulse sequences can cause auditory activation (Braun et al., 2020; Guo et al., 2018; Sato et al., 2018). However, due to the long delay between stimulation and data collection, offline effects cannot be explained by an auditory artifact (Verhagen et al., 2019). For online protocols, an auditory mask can be used to avoid auditory confounds (Braun et al., 2020).

The tissue-effects of low-intensity FUS can be monitored online by thermometry and acoustic radiation force (ARF) imaging. ARF causes displacement of objects, including biological tissue, in the path of the ultrasound wave. This displacement can be detected by MRI using short pulses of ultrasound synchronized to a motion-encoding gradient (de Bever et al., 2018; McDannold and Maier, 2008; Paquin et al., 2013). Additionally, thermal mapping can be achieved with dedicated MR sequences (Cline et al., 1994; Parker et al., 1983). Using simultaneous ARF imaging and MR thermometry, Ozenne and colleagues (2020) have shown that changes in temperature at the focal point with low intensity FUS were minimal. With a 0.85 MHz transducer, they also showed that temperature at the skull could increase up to 2 °C.

#### 7.1.2 Applications of FUS for behavioral and neural modulation

At the neuronal level, a 100 ms burst of ultrasound targeting the FEF evokes transient excitatory and inhibitory modulation of neuronal activity in the adjacent supplementary eye field (SEF) (Wattiez et al., 2017). At the network level, resting-state MRI has been used to assess offline FUS effects on the coupling between the stimulated area (e.g. the medial frontal cortex, the somatosensory cortex, the amygdala, or the striatum) and distant regions (Folloni et al., 2019; Khalighinejad et al., 2020; Verhagen et al., 2019; Yang et al., 2018) (Fig. 8 B). When stimulation bursts are repeated over up to a 40 s period, neuromodulatory effects are observed offline for durations of 10 to 90 min (Dallapiazza et al., 2018; Folloni et al., 2019; Khalighinejad et al., 2020; Pouget et al., 2020; Verhagen et al., 2019). In vitro results suggest that TUS effects could even last for up to 8–14 h (Clennell et al., 2021). The in-vivo modulation that was observed included increased coupling with multiple areas strongly coupled with the target area, both superficial and deep (with the exception of the basal forebrain). Importantly, effects of transcranial FUS are regionally specific. Each brain area can be characterized by a specific set of inputs and outputs called a connectivity fingerprint. Sonication of distinct regions of the medial frontal cortex caused changes in each area’s connectivity fingerprint, but only when sonication was applied to the area itself (Verhagen et al. 2019). For example, the amygdala and ACC are part of each others’ connectional fingerprints and so when FUS was applied to either ACC or amygdala it affected coupling between the two areas but only the application of FUS to ACC affected the rest of ACC’s connectional fingerprint and only the application of FUS to amygdala affected the rest of the amygdala’s connectional fingerprint (Folloni et al., 2019). Finally, sonication of deep structures can be done without impacting the superficial cortex that lies in the path of the ultrasound beam. For instance, stimulation of the amygdala can be conducted without observing changes to the functional coupling of cortical areas around the superior temporal sulcus (Folloni et al., 2019).

FUS-induced changes in neural activity also affect behavior. A unilateral single FUS burst over the FEF slowed the latency of antisaccades made towards the hemifield ipsilateral to the stimulation site (Deffieux et al., 2013). Unlike reflexive saccades, which are made towards flashing visual stimuli, antisaccades are made in the opposite direction and are thought to index the volitional or intentional control of eye movements; a large body of work examining FEF lesions and inactivation effects suggest that antisaccades are particularly vulnerable when FEF function is compromised (Pierrot-Deseilligny et al., 2003) and so the FUS results are consistent with these findings. With a similar stimulation protocol, Kubanek and colleagues (Kubanek et al., 2020) recently showed that FEF stimulation could bias the primates’ decisions in a two-alternative choice task of intentional eye movement control. Offline stimulation protocols have also proven to be useful to parse the contributions of brain areas to decision-making processes. Bilateral stimulation of the anterior cingulate cortex altered the ability of NHPs to hold evaluation of counterfactual choice options (i.e., choices that are not being taken right now but which might be taken in the future) and to translate them into behavioral changes on subsequent trials in a reward-guided decision making task (Fouragnan et al., 2019; Khalighinejad et al., 2020; Kubanek et al., 2020). Basal forebrain stimulation might not change which decision is taken but it does alter the timing of the decision (Khalighinejad et al., 2020). Recent work in rodents demonstrated the feasibility of conducting FUS experiments in freely-moving animals (Lee et al., 2018), an option that could also be explored in future NHP studies.

#### 7.1.3 Applications of FUS for targeted drug delivery and blood-brain-barrier opening

Focused ultrasound (FUS) can also be used in conjunction with microbubbles to deliver drugs to specific brain targets (Fig. 8 A,C). This is accomplished by combining FUS with intravenous injection of lipid microbubbles to increase the permeability of the blood-brain barrier (BBB) (Hynynen et al., 2005; Tung et al., 2011). BBB permeability can remain elevated for 48-72 h (Marquet et al., 2014), allowing molecules that are too large to cross the intact BBB to enter the brain. Entry is limited to the region in which the BBB is opened, thus improving the targeting specificity of drug delivery and potentially reducing undesirable side effects. BBB opening has been performed in awake, behaving NHPs engaged in a decision-making task without interrupting their performance, and with no signs of pain or distress (Downs et al., 2015b, a) (Fig. 8 C and D). When tested hours after BBB opening, NHPs showed significant improvements in decision-making performance (Downs et al., 2017), demonstrating that the BBB can be opened without sedation or anesthesia. In NHPs, it has been established that the volume of brain within which BBB permeability is increased depends on FUS pressure and can range from less than 200 mm^3^ to over 800 mm^3^ (Samiotaki et al., 2017). The amount of drug delivered, as well as the kinetics of transport, also scale with FUS pressure.

Ultrasound induced BBB permeability changes have recently been exploited to allow the delivery of neuroactive agents to produce localized modulation of brain activity. Opening the BBB has been shown to facilitate the blockade of neural activity by the inhibitory neurotransmitter GABA in the somatosensory cortex of both rats (McDannold et al., 2015) and NHPs (Constans et al., 2020). GABA does not cross the intact BBB. The effects of GABA were tested by measuring suppression of somatosensory evoked potentials (SSEPs) which scaled linearly with GABA dosage in both studies. The suppression lasted up to two hours after a single bolus injection. No suppression was seen without BBB opening. In addition to delivery of conventional drugs, BBB opening via FUS with microbubbles allows the delivery of viral vectors for gene transfer, as used in optogenetics or DREADDs investigations (see Sections 4 and 6 above). FUS-mediated BBB opening has been used to deliver AAV-GFP (Hsu, 2013; Thevenot et al., 2012; Wang et al., 2015a,b), AAV-LacZ (Alonso et al., 2013), AAV-channelrhodopsin-2 (Wang et al., 2017), and DREADDs (Szablowski et al., 2018). As an alternative to viral gene delivery, Mead et al. (2016) administered DNA-bearing nanoparticles coated with polyethylene glycol, thus avoiding immune responses against the virus. They tested this by delivering the gene for glial-derived neurotrophic factor (GDNF) to the striatum in a rat model of Parkinson’s disease (Mead et al., 2017).

Further studies are needed to fine-tune the spatial targeting, extent, degree, and duration of BBB opening as a function of FUS parameters. For neural circuit-tracing studies, it is desirable to deliver a high concentration of drug within a small volume of tissue (e.g. a single cortical layer) with sub-millimeter targeting accuracy. Achieving this is likely to require a precise 3D model of the subject’s skull, as well as computational methods that model the interaction of the FUS beam with the skull and soft tissues (Karakatsani et al., 2017 Younan et al., 2013a,b).

### 7.2 Discussion and outlook

The general advantage of ultrasound neurotechnologies is their non-invasiveness. This offers a more straightforward path to many more human applications. However, risks for human and non-human primate application must be considered carefully and specifically for each type of ultrasound method. Because high-intensity FUS leads to thermally-induced brain lesions, approaches for non-clinical use tend to focus on low-intensity FUS. Most neuromodulation protocols used for low- intensity online and offline FUS have not been shown to induce any tissue trauma (Blackmore et al., 2019; Gaur et al., 2020; Verhagen et al., 2019). However, one protocol that used a very high number of repeated low-intensity stimulations (*n* = 600) in sheep induced microbleeds in 50% of the animals studied (Lee et al., 2016b). Therefore, FUS protocols should be designed and implemented very carefully. MR thermometry has shown that with low intensity FUS, induced changes in temperature at the focal point of stimulation were minimal (Ozenne et al., 2020), but at higher intensity-usually applied for conducting controlled brain lesions in patients (Bretsztajn and Gedroyc, 2018; Elias et al., 2016)-temperatures could rise above 50 °C, as required for clinical thermal ablation. Finally, it should be noted that the higher the frequency, the more energy the skull absorbs, which could both impact the dura or neural tissue that lies just underneath it and cause unintended side effects and/or prevent intended effects on deep brain regions from materializing. Therefore, choosing a transducer that can generate high frequency FUS with the aim of achieving more focal stimulation could be counterproductive for the intended purposes. Computational modeling of the pressure wave in the brain can be used to improve targeting accuracy and safety (Wu et al., 2018). Although a few studies have already been conducted on human subjects (Braun et al., 2020; Legon et al., 2014; Reznik et al., 2020) work with animal models remains essential for understanding TUS mechanisms, establishing safety limits and developing safe new stimulation protocols. In addition, in animal models TUS can be readily combined with invasive recording techniques to provide new insights on neuronal computations, mechanisms and circuits.

FUS-mediated BBB opening is associated with other specific considerations. Concerns over potential immune responses (Kovacs et al., 2017) have been noted at high microbubble concentration levels (Hynynen et al. 2017). These can be mitigated by using FUS at low (clinically or non-clinically approved) microbubble concentrations and/or to release drugs sequestered in micelles, liposomes, or nanoparticles activated at frequencies and intensities that do not disrupt the BBB (Husseini et al., 2014). Wu et al. (2018) found that octaflouropropane and de-caflourobutane nanodroplets successfully delivered 40 kDa molecules (dextran) with FUS at pressures of 300 and 450 kPa without cavitation damage. To avoid drug effects at untargeted locations, nanoparticles composed of biodegradable and biocompatible constituents allow noninvasive uncaging of a neuromodulatory drug (Airan et al., 2017). Overall, the proliferation of different methods for neuromodulation and drug delivery, combined with precise modeling of how FUS interacts with biological tissues at different frequencies and pressures, and emerging methods for online monitoring of pressure and temperature inside the brain should lead to safer and more effective FUS protocols.

## 8 Infrared neural stimulation

### 8.1 Infrared neural stimulation (INS) and neuroimaging

**Fig. 9:**
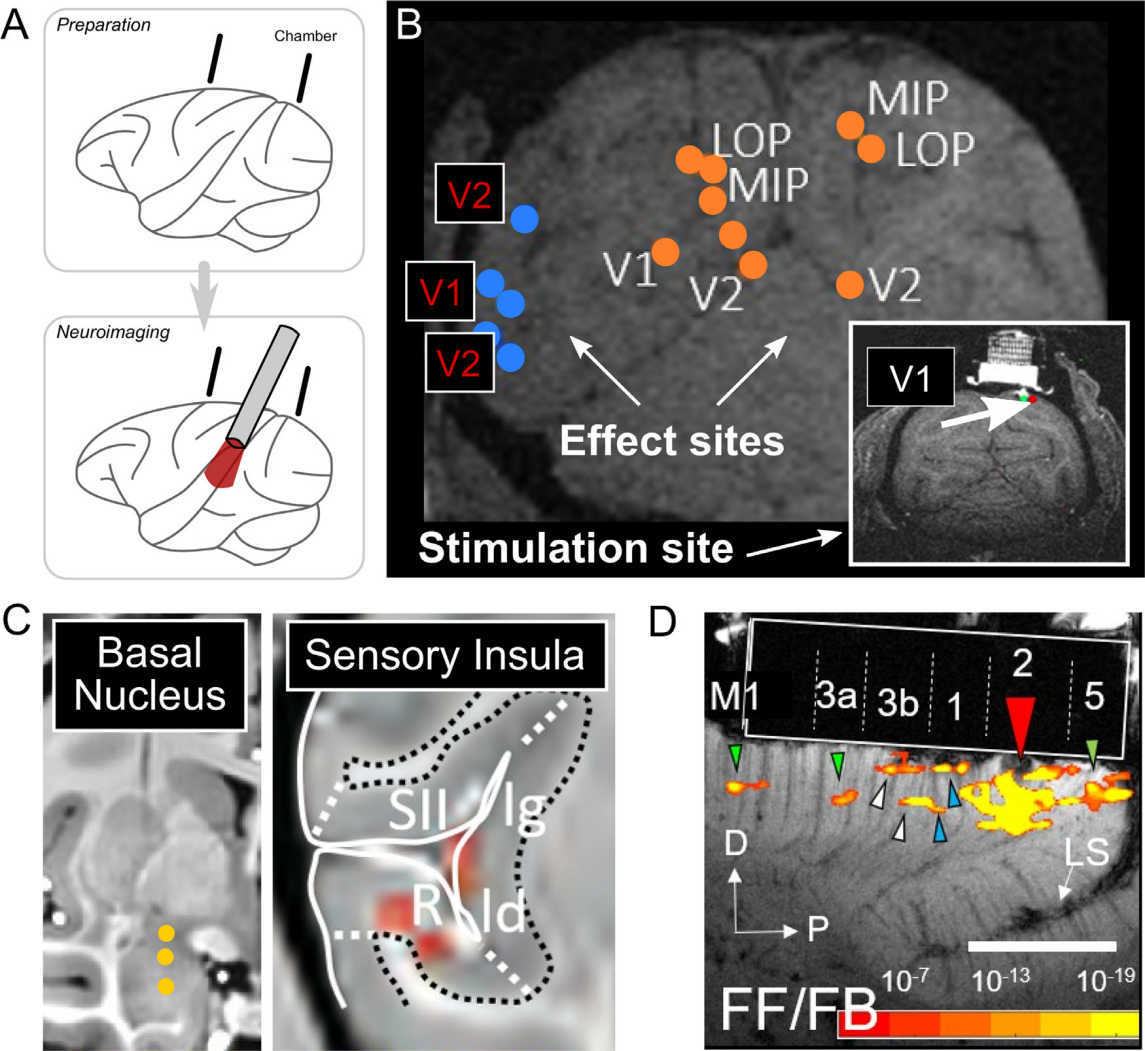
Infrared Neural Stimulation (INS) and neuroimaging. A) Schematic of the technique. After implantation of a recording chamber, optic fibers are used to focally stimulate the brain with pulsed infrared light. B) Cortical stimulation in V1 via an optic fiber that is inserted through a grid in a recording chamber produces focal activation at the fiber tip (inset, green) as well as a nearby spot of cortex close to the fiber tip (inset, red), consistent with optical imaging findings (Cayce et al 2014b). Other activated sites in the visual cortex are depicted with blue and orange dots. C) Subcortical stimulation in the basal nucleus of the amygdala (left, yellow dots) evokes focal activations in the sensory insula (lg, ld), auditory (R), and somatosensory (SII, in adjacent slice) cortices. Adapted from (Xu et al., this issue). D) Feedforward vs feedback connections. Top: Stimulation of a single digit site in squirrel monkey somatosensory cortex (SI) with the fiber tip in Area 2. Feedforward (FF) effects are found as middle layer activations in M1 and 3a. Bilaminar feedback (FB) activations are seen in 3b, and 1. (LS: lateral sulcus, D: dorsal, P: posterior). Adapted from Xu et al. (2019).

A brain perturbation technique called infrared neural stimulation (INS) has recently been developed for fMRI (Xu et al., 2019). This optical method employs a long wavelength laser light (1875 nm) to focally stimulate a point in the brain via brief pulses of optically induced heat transients and can readily be combined with fMRI (Fig. 9 A). At optimal wavelengths for optical energy absorption by water, rapid heat transients are capable of inducing action potentials and tissue penetration is both effective and focal (~300 pm into tissue) at a wavelength of ~1.9 micron (Wells et al., 2005). Due to the briefness of the optical pulse (250 microseconds), there is time for heat to dissipate, avoiding tissue damage. The application of INS to rat sciatic nerves furthermore evokes action potentials and results in muscle contractions (Wells et al., 2007). While the biophysical mechanism of INS in mammalian neural tissue is still under study, the most likely mechanism is the induction of transient membrane capacitance changes that lead to action potentials (Plaksin et al., 2018; Shapiro et al., 2012).

There are currently several exciting applications of INS, including cochlear stimulation for treatment of deafness (Tan et al., 2018) stimulation of the heart for cardiac pacemaking (Ford et al., 2018), inhibition of action potential propagation in fine fibers involved in pain perception (Ganguly et al., 2019), and suppression of action potential generation at neuromuscular junctions (Zhu et al., 2019). Here, we will focus on neuroimaging applications in NHPs.

#### 8.1.1 INS for mapping brain circuits at mesoscale and for behavioral modulation

The first use of the INS method in the central nervous system was conducted by Roe and colleagues (Chernov and Roe, 2014). Optimal parameters for cortical stimulation (0.5 sec pulse train, 250 μs/pulse, 200 Hz, 1875 nm) were first determined with systematic studies in rat cortex, where INS evoked action potentials and hemodynamic responses (intrinsic signal or ‘initial dip’) similar to that evoked by sensory stimulation (Cayce et al., 2011). These cortical responses were both reliable and intensity dependent. Calcium dynamics of neurons and astrocytes were also shown to be modulated by in vivo INS stimulation (Cayce et al., 2014a). This groundwork opened doors for developing INS as a tool for functional modulation and circuit mapping.

The accurate mapping of functionally specific columnar circuits in the living primate brain is challenging due to the small size of cortical columns (typically 200-500 pm), and the fact that traditional methods of anatomical tract tracing for long range connections (typically 2-5 mm sized injections) or standard resolution DTI (typically 2–3 mm voxels) often lack submillimeter resolution. To overcome this challenge, INS was first tested with optical imaging experiments in monkey visual cortex where columnar organization and circuitry are well known. Using a 200 pm optic fiber to stimulate cortical columns while imaging the evoked cortical response, Cayce et al (Cayce et al., 2014b) showed that the action potential inducing effect of INS stimulation was intensity dependent and confined to single ocular dominance columns. It also caused selective activation of nearby eye-matched columns, demonstrating that much like electrical (Friedman et al., 2020; Hu et al., 2020) and optogenetic (Chernov et al., 2018) stimulation (see Sections 5 and 6 ibove), INS of single cortical columns leads to the activation of connected columns (e.g., orientation domains, ocular dominance domains) in primate cortex.

Combining INS with ultrahigh-field MRI (INS-fMRI) allows detailed mesoscopic network mapping at the whole brain level (Xu et al., 2019). For example, stimulation in V1 of cat and monkey produces expected activations in cortical (primary and higher order visual cortical areas) as well as subcortical (LGN, pulvinar) locations (Fig. 9 B). These maps replicate known anatomical visual pathways and provide a whole-brain view of the network associated with a single column. Depending on stimulation intensity and signal-to-noise, both monosynaptic and disynaptic connections are revealed. Perhaps the most exciting aspect of this INS-fMRI method is the immediacy and speed of the mapping procedure. Visualization of whole-brain networks is possible online during data acquisition, and whole-brain networks can be obtained within single MRI sessions.

Subcortical sites can also be systematically stimulated by insertion of optic fibers into deep structures. For example, the connections of multiple sites within the basal nucleus of the macaque amygdala were studied and compared, revealing focal and patchy activations at connected sites in the insula and the cingulate cortex (Fig. 9 C) (Xu et al., 2021) this both confirms and extends previous anatomical studies of amygdala connectivity. In addition, if mapping is conducted with sufficiently high resolution to reveal cortical laminar definition, one can distinguish feedforward vs feedback pathways. This is illustrated in Fig. 9 D, where stimulation of area 2 in monkey somatosensory cortex (SI) produces 200 pm-sized middle layer activation (feedforward) in areas M1 and 3a, and bilaminar activation (feedback) in areas 3b and 1, a finding consistent with known anatomical interareal connectivity in SI.

Behavioral modulation by INS has also been demonstrated. Targeting INS to visuotopically mapped cortical sites in awake behaving monkeys evokes highly reproducible saccades (location and latency), a result consistent with the induction of phosphenes (Roe et al., 2015). For longterm behavioral studies, it is important to ensure that repeated stimulation does not cause tissue damage. To evaluate this, a range of INS intensities was applied to rat and monkey cortex, the tissue assessed histologically (laminar profiles viewed in Nissl stains), and damage thresholds identified (Chernov and Roe, 2014). Using conservative intensity levels, repeated stimulation (hundreds of trials per session over months) revealed no adverse effects on neural signals or animal health (Roe et al., 2015).

### 8.2 Discussion and outlook

The optical nature of this method makes INS-fMRI easily amenable to the MRI environment. There are no complications with current spread, permitting focal column-specific or subnuclear-specific stimulation. The distinction between laser-fMRI (INS) and opto-fMRI (Gerits et al., 2012; Ohayon et al., 2013) is that laser-fMRI does not depend on viral transfection and thus can be delivered anywhere in the brain without prior injections. This ease of use is quite important for NHP work where viral transfection is more cumbersome (Tremblay et al., 2020). While INS is not cell type-specific, it is known that most long-range cortico-cortical connections in the brain are mediated by pyramidal neurons and, in that sense, some degree of cellular specificity is achieved for mapping large scale networks. Like other brain perturbation methods, and in contrast with anatomical tracing, INS provides insight into many distinct in vivo functional networks at the whole-brain level within single individuals.

There are multiple potential uses of this mapping method. The development of large-scale columnar connectomes in primates is now possible (Roe, 2019; Roe et al., 2020). For computational studies, understanding the topography of brainwide connections at mesoscale levels may lead to new insights in connectional topology, and neurophysiologists may target activated nodes, some previously unknown, for electrophysiological study. As INS is also capable of inducing behavioral modulation (Roe et al., 2015; Tan et al., 2018), the ability to link behavior with underlying brain networks makes it attractive for neurobehavioral and brain-machine interface applications. The ability to probe circuits repeatedly within single individuals also opens doors for studying changes in mesoscopic circuits over time (e.g. for development or aging studies). In addition, as this method circumvents the need for viral transfection, there is exciting potential for translation to human clinical application.

## 9 Directional and pathway-selective approaches

All perturbation tools discussed so far focused on experimenter-induced (in)activation of a specific cortical or subcortical target, with the aim to assess its causal contribution to perception, cognition, action, and activity in other parts of the brain. Such procedures, however, can impinge upon all of an area’s out- and ingoing connections and fail to discriminate between different circuits intersecting at the targeted brain area. Hence, in the behavioral or functional effects caused by virtually all causal interventions, an area may be affected by artificially altered activity in any or all of these intersecting circuits. Therefore, to better isolate the causal contribution of a specific pathway in a perceptual, cognitive or motor task, one should specifically (in)activate activity within the target circuit only, without affecting others.

Although pathway-selective perturbation tools are mainstream in rodent research (Tye and Deisseroth, 2012), they have not yet been used in primates much. One pioneering study achieved reversible pathway-selective inactivation in the spinal cord of the macaque by injecting a highly efficient retrograde lentiviral vector (HiRet) in a target region of projection cells and another adeno-associated virus vector (AAV2) in the region housing the cell bodies of these projection neurons (Kinoshita et al., 2012). The retrograde vector carried a gene for an enhanced tetanus neurotoxin (eTeNT) downstream of a tetracyclineresponsive element (TRE). The AAV vector carried a reverse tetracycline transactivator (rtAV16). Hence, the eTeNT is conditionally expressed only in double-labeled neurons as it requires the transactivator. Moreover, only in the presence of doxycycline (a tetracycline), eTeNT will suppress synaptic transmission restricted to the double-infected projection neurons. In this first study, the technique revealed the critical role of propriospinal neurons in the control of hand dexterity in macaques. The same powerful double-infection technique was also instrumental to reveal the critical role of the connections between the superior colliculus and the ventrolateral pulvinar in blindsight after a V1 lesion (Kinoshita et al., 2019). Reversible inactivation of this pathway impaired visually guided saccades to the contralateral hemifield of blindsight monkeys.

In another recent study, the technique was used in combination with functional imaging in monkeys performing cognitive tasks (Vancraeyenest et al., 2020). Specifically, the authors reversibly inactivated the connections originating in the ventral tegmental area (VTA) and projecting to the nucleus accumbens (NAc) in monkeys. This predominantly dopaminergic pathway purportedly plays an important role in reinforcement-based learning. Contrary to this prediction, however, doxycycline-induced inactivation of the VTA-to-NAc pathway yielded no effect on reversal learning during an object reversal discrimination task. Instead, motivational behavior requiring high effort levels was severely diminished in another task. Moreover, reversible inactivation of the connections between the VTA and NAc resulted mainly in increased functional connectivity, measured with resting state fMRI, primarily between temporal and frontal regions. In the Vancraeyenest study, both HiRet (a vector pseudotyped with the fusion glycoprotein type B (FuG-B)) and NeuRet (with the fusion glycoprotein type C (FuG-C)) were used as retrograde gene transfer vectors. While both lentiviral vectors are highly efficient retrograde gene transfer vectors in primates, NeuRet possesses higher neuronal specificity than the HiRet vector (Kato et al., 2011). Recently, an even more efficient neuron-specific retrograde vector has been developed using another type of fusion glycoprotein (FuGE) (Tanabe et al., 2017).

### 9.1 Discussion and outlook

The development of highly efficient retrograde transfer vectors in combination with conditional expression of genes to downregulate (e.g., the aforementioned neurotoxins or hyperpolarizing opsins) or enhance activity (e.g., in combination with depolarizing opsins) will offer fascinating new tools to dissect the contribution of specific neural pathways in perception and cognition (Inoue et al., 2015; Kinoshita et al., 2019, 2012; Nurminen et al., 2018; Tohyama et al., 2017). For translational purposes, these novel pathway-selective interventional approaches may have benefits over conventional region-specific methods, as they may mitigate unwanted side effects caused by the (in)activation of intersecting circuits at a target site that are not relevant for treatment of the disease.

Major challenges related to pathway-selective genetic based perturbation methods are highly similar to those described for optogenetics (see Section 6.3). It remains challenging to restrict diffusion of viral vectors exclusively to the intended targets, as is the case for all types of injections in the brain, including tract-tracing molecules, drugs, etc. A key control to reduce off-target effects is to combine the injection procedures with the most refined in-vivo imaging methods, for example using MRI while co-injecting MnCl_2_ or GD-based contrast agents, or by combining injections with simultaneous electrophysiological recordings, or both.

The method can be used for studying the functionality of specific pathways at a mesoscale level, such as investigating functional interactions between blobs in area V1 and different stripe compartments in area V2, provided the dual injections can be confined to these specific functional subcompartments. This will most likely require the use of optical imaging or focal ultrasound imaging methods to identify the exact targets (i.e., in the example above, those would be the blobs in V1 and a specific stripe compartment in V2). At macroscale levels, the technology can be used to investigate functional interactions across areas, such as interactions between the FEF and area V4. Both the meso- and macroscale applications can be rendered cell-type specific when additional promoters in the vector are inserted to target these cell types (e.g., TH promoter to target dopaminergic neurons). An obvious limitation is that viral vectors which are cell-type selective and also efficient to use, are currently still very limited in availability and not used much for NHP research.

Theoretically, the reversible pathway-selective perturbation methods described above would be applicable for human use, since viral vector-based treatments are currently already implemented in a variety of clinical applications. Obvious safety protocols should be followed, as is the case with all clinical viral vector methods. The advantage of the dual injection technique compared to, for example, optogenetics is that after injection of the viral vectors, no additional invasive procedures are required to ‘activate’ or ‘inactivate’ the targeted pathway. Because of the presence of a reverse transactivator in combination with the tetracycline-responsive element, only a tetracycline needs to be administered (e.g. orally) to activate the downstream gene (in the example above, this would be eTeNT). It is conceivable to insert different genes downstream relative to the tetracycline-responsive element, whereby a specific pathway can be reversibly boosted instead of silenced, as long as the tetracycline is given to the patient. Such pathway-selective clinical interventions would offer the possibility to focally target (inactivate or activate) a specific pathway within a complex system affected by a specific disease and then assess improvement in symptoms. As an example, this approach could be used to evaluate an under- or over-performing dopaminergic reward system in the case of depression or addiction, respectively. The approach could be more powerful and induce fewer side effects because it would not impact off-target pathways that may be highly relevant to support normal behavior unaffected by the disease.

## 10 General discussion

Brain perturbation methods are vital for advancing understanding about the causal mechanisms of distributed brain functions. Employing brain perturbation techniques in NHPs has been and will continue to be instrumental for scientific and biomedical advances. Combined with neuroimaging, these techniques and the insights gained with them can become even more powerful, allowing brain-wide visualisation of the perturbation impact on brain systems and interconnectivity. Neuroimaging also allows comparison of effects across methodologically distinct techniques. Such comparisons enhance insights into the mode of action of the perturbation techniques, which is vital for understanding how they can optimally translate to and benefit humans. The interplay between neuroimaging and brain perturbation not only improves our understanding of the brain perturbation techniques, but it can also provide novel insights into the neural underpinnings of the neuroimaging signals themselves (e.g., the MRI-BOLD signal).

This review could not cover every brain perturbation technique that has been used in combination with neuroimaging. It focuses on several of the more commonly used techniques that are regularly combined with neuroimaging, and highlights some new approaches with exciting potential. Other brain perturbation approaches like cooling (Khachaturian et al., 2008), Transcranial Magnetic Stimulation (TMS) (Balan et al., 2017), or transcranial current stimulation (tDCS/tACS) (see Liu et al., 2018), and references therein) are less commonly used in NHPs, even though they are regularly combined with neuroimaging in humans (e.g., TMS-fMRI). These methods may be less commonly studied in NHPs for both practical reasons and because of the greater specificity of other approaches. Nonetheless, even for these or other approaches, work with NHPs may be required to understand the underlying neural mechanisms and the combination with neuroimaging could be an important component (Balan et al., 2017).

This paper offers a snapshot of where the field stands in terms of NHP brain perturbation and neuroimaging advances. However, because many developments are ongoing, we envision a dynamic resource on brain perturbation and neuroimaging that evolves along with advances in the field as a complementary resource to this paper. The PRIME-RE platform (Messinger et al., 2021), that was recently launched by members of the PRIME-DE community as an online research exchange platform for all things related to NHP neuroimaging. It offers an excellent outlet for such dynamically evolving content and will encourage data sharing of legacy and new data, as well as offering primers to support laboratories to use and develop the techniques further. Maintained by the community, researchers can upload technique primers, tips, tricks, risks, solutions and warnings for all relevant methodologies. This ongoing effort will help the scientific community to keep up to date with recent developments and to support researchers aiming to implement the techniques in their own labs. PRIME-RE also maintains collections of links to open NHP neuroimaging datasets and could facilitate the distribution and accessibility of valuable perturbation datasets, that might for instance be hosted on PRIME-DE (Milham et al., 2018).

### 10.1 Anesthesia as a perturbation method

The implications of performing brain perturbation and neuroimaging studies in either anesthetized or awake animals were briefly mentioned here and more extensively detailed for NHP studies elsewhere (Basso et al., 2021). However, anesthesia has non-uniform effects on the brain, and different anesthetic agents impact different systems in the brain (e.g., pain neuraxis, consciousness). For instance, anesthesia suppresses the feedback of neural processing (Suzuki and Larkum, 2020), and some brain regions, such as the precuneus, posterior cingulate gyrus, prefrontal dorsolateral cortex and thalamus, are more strongly deactivated by various forms of anesthesia than other regions (Franks, 2008). The non-uniform effects of anesthesia have been exploited in studies on the neural mechanisms of auditory sequence violations (Bekinschtein et al., 2009). For instance, neural responses to oddball sounds (local violations) primarily engage earlier auditory processing stages, while relationships that require between sequences integration of information (global violations) engage broader brain circuits, including the prefrontal, parietal and cingulate cortices (Uhrig et al., 2014). The local violation effect in the auditory cortex disappears under propofol anesthesia (a GABA-ergic agonist) and, interestingly, shifts spatially under ketamine anesthesia (an NMDA antagonist) (Uhrig et al., 2016). A progressive disorganization of the global effect in the prefrontal, parietal and cingulate cortex was observed with increasing levels of propofol anesthesia, while ketamine completely suppressed these same areas. Anesthesia has also been shown to massively reconfigure dynamic resting-state networks (Barttfeld et al., 2015; Uhrig et al., 2018) despite a preservation of stationary resting-state networks (Vincent et al., 2007). Although anesthesia is not usually thought of as a brain perturbation approach, using neuroimaging to understand anesthetic agent mechanisms and visualize the impact on the brain is important scientifically as well as for clinical and other translational reasons.

### 10.2 The perturbed brain as a functional model

One important limitation of perturbation studies is that adding or removing activity may force the brain into a state that might not occur naturally. On the other hand, brain impact or disease is by definition not normal. Thus, one could question to what extent the resulting effects are representative of the brain’s normal mode of operation. In a sense, this question is not categorically different from questioning whether artificial stimuli employed in a laboratory environment can teach us anything about how the brain operates in natural environments. In this case, the input is much more complex, comprehensive and likely to simultaneously engage many processing networks, leading to a broad range of complex neural interactions (Rust and Movshon, 2005). Any precisely targeted brain perturbation is likely to modulate only a fraction of the networks typically involved in ‘natural’ perception, cognition, and behavior. As a consequence, drawing causal conclusions at the systems level after perturbation of a single node in a complex network should be done with caution, especially when asserting necessity or sufficiency. Future NHP studies could also perturb multiple nodes in the network to more rigorously and thoroughly assess causal relationships (Qiao et al., 2020), a unique approach that is not possible with humans. However, a reductionist approach with either sensory stimulation or brain perturbation can be an advantage for teasing apart the mechanisms of a complex system that is the brain; so long as we remain aware of the fact that we are introducing a potentially unnatural brain perturbation. On the other hand, artificial stimuli can be designed to emulate natural conditions and likewise, with increasing understanding of their underlying mechanisms, the propagation of brain perturbation effects can become increasingly similar to natural signal propagation. In this regard, the spatio-temporal scope of the intended perturbation approach (Fig. 1) will allow selecting the most useful tool to use for the intended purpose. Careful comparison of brain dynamics in unperturbed and perturbed situations furthermore offers the advantage of studying the system in a more natural state or during perturbation, as natural or unnatural as that might be. After all, brain damage in patients is akin to a brain perturbation technique that disrupts normal functioning, with the experimental approach in NHPs as a model system having the advantage of being able to perturb the brain in a highly controlled way. Thus, although this paper underscores the value of brain perturbation approaches, the benefits of comparing results to descriptive studies without perturbation should not be dismissed.

### 10.3 Causality and comparison across perturbation techniques

We have emphasized that a key benefit of brain perturbation approaches is that they allow inferences on cause and effect. The various techniques can provide different, partially non-overlapping ranges of spatial and temporal resolution, and cell type- or pathway-specificity (Fig. 1), but there are several conditions that need to be met to obtain true causal information (Clark et al., 2011). As discussed in the technique-specific sections above, the confidence with which causality can be inferred depends on the level of understanding of the mechanism of action of a particular technique as well as its spatio-temporal scope. Even for techniques for which there is a substantial evidence base, this type of knowledge is still incomplete. A second limitation of any perturbation study is that even if a causal relationship can be asserted with relative confidence, it does not necessarily mean that this specific causal relation is sufficient or necessary to support a brain function or behavior (see also Section 10.2). A thorough comparison across different studies and perturbation methods, as well as approaches in which multiple nodes are perturbed simultaneously, may also shed light on this issue. Thus, it is crucial to continue to increase our understanding of the specificity and modes of action of individual perturbation approaches, as well as the strengths and limitations of different neuroimaging methods. Using neuroimaging to both record and compare the impact of different brain perturbation techniques will continue to shed light on the different modes of action and the types of causal inferences that can be made.

### 10.4 Suppressed or negative imaging signals

Functional imaging studies often tend to focus on positive brain signals, but neural processes can manifest as either increases or decreases of the neuroimaging signal. This is not unique to fMRI imaging but also applies to other neuroimaging modalities such as PET. Although baseline levels of brain activity can fluctuate and be difficult to define, both positive and negative effects can be crucial for understanding the neural mechanisms and mode of action of a brain perturbation approach. Yet, to rectify what may be a bias towards reporting positive responses, it is important to consider the neural basis of negative imaging signals. Intuitively, they could be related to neuronal suppression, but in some cases they could also reflect an entirely different process, which may be specific to the mode of action of a particular technique.

Sensory stimulation leads to reliable positive and negative fMRI BOLD responses (PBRs and NBRs) during topographical mapping (Allison et al., 2000; Fracasso et al., 2018; Harel et al., 2002 Klink et al., 2020). NBRs can occur next to PBRs in the visual cortex and have been shown to correlate with electrophysiologically-measured neuronal inhibition (Huang et al., 1996 Shmuel et al., 2006, 2002). NBRs are also found with frequency mapping of the auditory cortex, adjacent to areas of PBRs (Ortiz-Rios et al., 2017 Petkov et al., 2006; Tanji et al., 2010). Local inhibitory lateral connections (Angelucci and Bullier, 2003) as well as distal excitatory and inhibitory interactions might play a role in NBR effects through a push/pull mechanism (Angelucci and Bressloff, 2006). Although positive BOLD or MION-based fMRI responses tend to predominate during cortical electrical microstimulation, suppression of fMRI responses appears to be common in the few available NHP studies with subcortical stimulation. The presence of NBRs may depend on stimulation frequency, animal state (e.g., awake or anesthetized) and the intrinsic properties of the stimulated region (Logothetis et al., 2010 Murayama et al., 2011; Murris et al., 2020; Sultan et al., 2011).

Some negative imaging signal effects are not due to neuronal response suppression, and neuronal response suppression can even result in a positive fMRI signal if it increases neurovascular demands (e.g., greater responses by inhibitory neurons that require a vascular response). The nature of negative fMRI signals can furthermore differ substantially between BOLD-based fMRI and MION contrast-agentbased approaches (Leite et al., 2002; Mandeville, 2012; Vanduffel et al., 2001). MION is more commonly used with macaque 3T imaging for its superior contrast to noise properties and because it is a cleaner measure of cerebral blood volume (CBV) than BOLD-fMRI. Yet, hemodynamic differences between BOLD- and MION-based fMRI could be exploited to further study the nature of neurovascular coupling. For instance, Yu et al. (2016) found that MION-CBV resulted in earlier onset responses from arterioles and BOLD-fMRI resulted in later onset responses from venules. With new opsin development allowing the manipulation of neuronal activity using inhibition (Li et al., 2019), and the perturbation of transsynaptic circuits (Tervo et al., 2016), optogenetics combined with functional imaging could be used to clarify the neural bases of positive and negative fMRI effects.

There may also be technique-specific reasons for negative imaging signals. For instance, in optogenetics studies, high-power photostimulation can generate artifactual negative responses that are related to the high absorption of light by the local neural tissue, thought to result from intravoxel temperature gradients changes and in a decrease of the T2* signal (Albers et al., 2019). This particular effect can partially be resolved by using red-shifted opsins which allow the use of longer wavelength light for stimulation (red instead of blue), which exhibits less tissue absorption and moderate scattering. Widespread distal negative responses have been reported in rodent studies of optogenetically stimulated cortex (Chan et al., 2017), but the neuronal bases for such effects require further study and a corresponding primate optogenetics study.

Neuronal recordings and high-resolution imaging could help to illuminate the bases for both positive and negative imaging effects. With the increasingly regular use of higher magnetic field strength MRI scanners in humans, it is now becoming possible to functionally differentiate signals from different sets of cortical layers in both humans (Huber et al., 2020, 2014) and animals (Chen et al., 2019a,b), and it is evident that MION-CBV and BOLD-fMRI responses can pick up different signals across the cortical layers (Goense et al., 2012; Jin and Kim, 2008; Smirnakis et al., 2007). Although highly ambitious, future high field perturbation neuroimaging studies combined with laminar neurophysiological recordings could fundamentally improve our understanding of brain systems and neural mechanisms of brain perturbation effects.

### 10.5 Translational potential rooted in a solid fundamental science foundation

Perturbation techniques in NHPs have substantial translational potential. This is discussed at length for the different techniques in the Discussion and Outlook sections above, but we will briefly summarize and present an overview here. The obtained knowledge about causal brain mechanisms and the development of safe and effective methodologies could all inform and improve treatment options in humans. Some perturbation approaches complement methodologies that are already being used in humans and provide vital information that would be very difficult or impossible to obtain in humans. Other approaches may soon have greater use in humans.

The development of DBS in humans as a treatment option for debilitating disorders substantially benefited from electrical stimulation studies in NHPs and will continue to do so as further information on mode of action and brain impact remains needed. Now that DBS is also being used to treat neuropsychiatric disorders, or to enhance pharmacological treatment, the combination of neuroimaging and electrical/pharmacological perturbation in controlled NHP studies is an indispensable part of the translational pipeline. Its study in NHPs could maximize clinical translational potential and benefits, give rise to successful translational pipelines from rodents to primates to humans, and incidentally, in the other direction as well.

Other brain perturbation approaches (e.g., permanent or reversible lesions, pharmacological and genetic manipulations) also have well-established links to human clinical research, while some of the newer techniques (e.g., ultrasound, infrared stimulation) are at different stages of translation to humans. For example, while FUS is already being used in humans, this application firmly depends on information that is being obtained with rodents and NHPs concerning its mode of action, impact on neural systems, and optimal parameters for effective and safe use (including blood-brain barrier manipulations).

Although translation to humans is an important goal of biomedical science, advancing scientific knowledge is an equally, if not an even more important objective. Moreover, clinical translation may simply not be possible, fail, or be unsafe for humans without a firm bedrock of knowledge provided by fundamental animal research. Comparative neuroimaging studies are an example of fundamental science that focuses primarily on advancing the kind of scientific knowledge that is crucial for understanding the evolution of neural systems and identifying evolutionary conservation and divergence across species. Such understanding is vital to take into account when translating scientific results and knowledge between animal model systems and humans (Mars et al., 2018; Van Essen and Dierker, 2007; Xu et al., 2020a,b).

Another important limitation of any translational or preclinical study, including those involving brain perturbations, is the biological variability in both the (clinical) population and the sample of subjects or animals enrolled in the study. For NHP studies that are often performed with relatively small sample sizes for ethical or practical reasons, this becomes particularly challenging when results are projected to a heterogeneous clinical population of a poorly understood disorder. One partial solution to this challenge is to design studies that address basic functions that can be related to symptoms, but are only partial disease or disorder models. This approach is built around the assumption that basic functions and their biological substrates are less variable than dysfunctions or disorders, which are often diagnosed based on heterogeneous collections of symptoms. An improved understanding of a functioning system can thus provide the bedrock for designing or improving treatments for dysfunctional neural systems. This approach is also a core component of the Research Domain Criteria (RDoC) framework suggested for the study of mental disorders (Insel et al., 2010). The sharing and combining of datasets and methods is another way to increase sample sizes and facilitate meta-analyses. The PRIME-DE (Milham et al., 2018; The PRIMatE Data Exchange (PRIME-DE) Global Collaboration Workshop and Consortium et al., 2020) and PRIME-RE (Messinger et al., 2021) initiatives are specifically designed to facilitate these efforts for NHP neuroimaging research.

Finally, rather than assuming that any primate brain will be a good model for the human brain, basic science studies, including cross-species comparative neuroimaging, will continue to be needed to assess this assumption by explicitly testing for correspondence or divergence across species (Orban et al., 2004; Rocchi et al., 2021; Tootell et al., 2003; Vanduffel et al., 2014). For example, cross-species alignment of cortical receptor and gene expression revealed highly similar patterns of serotonin 5HT_1A_ receptors in humans and macaque monkeys, and weaker correspondence with rats (Froudist-Walsh et al., 2021). This is promising for NHP models of depression, as conventional antidepressants increase 5HT_1A_ receptor signalling (Carhart-Harris and Nutt, 2017). Moreover, growing evidence suggests that the neural substrate of some cognitive functions is mostly symmetrical across the two brain hemispheres in NHPs, whereas substantial hemispheric lateralization exists for similar functions in the human brain (Balezeau et al., 2020; Hutchison et al., 2012a). Such differences between NHPs and humans have been identified in hemispheric lateralization of the fronto-parietal networks involved in spatial attention (Kagan et al., 2010; Mantini et al., 2013; Patel et al., 2015; Wey et al., 2014). The identified differences between humans and NHPs should be better understood, and considered alongside the evidence for shared evolutionarily conserved principles of primate brain organization. As we have noted here, NHP research often converges with and informs work in human patients. For instance, the compensatory, beneficial involvement of the intact hemisphere after unilateral lesions in NHPs (Sections 2 and 3) is in line with patient studies that demonstrate the recruitment of the opposite hemisphere during recovery after unilateral brain damage (Bartolomeo and Thiebaut de Schotten, 2016; Umarova et al., 2016). Likewise, the role of areas along the superior temporal sulcus in spatial orienting and attention that emerges from NHP studies is paralleled by a recent emphasis on temporal and temporal-parietal damage in neglect patients (Karnath and Rorden, 2012).

The exquisite technological approaches, along with the corresponding expansion of knowledge they have generated, illustrate that the field of combined brain perturbation and neuroimaging in non-human primates carries tremendous promise for science and medicine. It has, and will continue to have, a distinct, important, and lasting influence on both fundamental and translational neuroscience.

## Figures and Tables

**Fig. B1:**
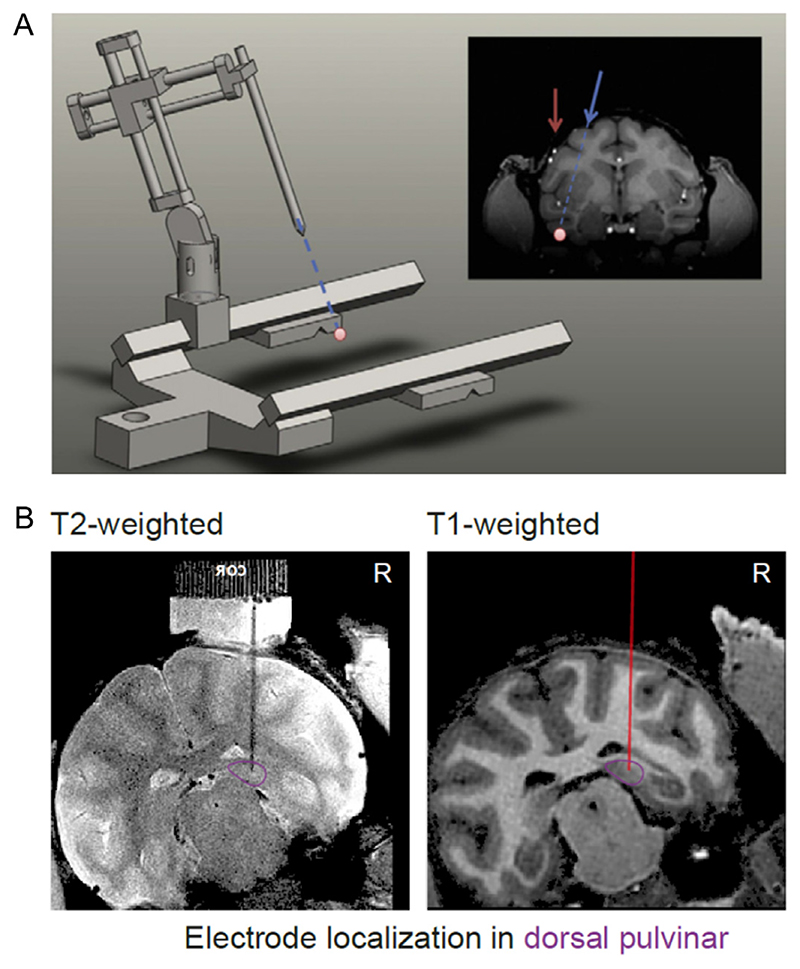
Targeting a part of the brain for perturbation. A) Targeting software (Planner) that allows the alignment of a stereotaxic manipulator with a target identified using MRI (pink dot). Reproduced from Ohayon and Tsao (2012). B) Pt-Ir electrodes can be localized using a T2-weighted scan and coregistered to a T1-weighted scan for more anatomical detail. Adapted from Dominguez-Vargas et al. (2017).

**Table 1:** Resources for targeting and peri/post-surgical localization.

Name	Link	Description
ElectroNav Toolbox	https://github.com/MonkeyGone2Heaven/ElectroNavToolbox	ElectroNav Toolbox is a collection of Matlab functions that are designed to assist with neurophysiological experiments involving acute electrode penetrations through implanted dural recording chambers.
Planner	https://github.com/shayo/Planner	Planner, a novel framework for MRI-stereotactic registration and chamber placement for precise electrode guidance to recording, stimulation and injection sites in MRI space. (Fig. B1A)
Cicerone	http://neuromod.umn.edu/downloads.html	Cicerone* is a stereotactic neurosurgical tool that combines visualizations of MRI, CT, and a 3D brain atlas with visualizations of microelectrode recording tracts for surgical planning in DBS lead placement. * incorporated by Boston Scientific into Guide™ DBS commercial software https://www.bostonscientific.com/en-EU/products/deep-brain-stimulation-systems/Guide-DBS.html
pyElectrode	https://github.com/pierredaye/pyElectrode	pyElectrode, visualization and planning of electrode sites and orthogonal chamber projections
Lead-DBS	https://www.lead-dbs.org/lead-dbs-optimized-to-support-macaque-imaging-data/	Lead-DBS is a Matlab toolbox built to localize deep brain stimulation electrode placements based on postoperative imaging data
Brainsight	https://www.rogue-research.com/veterinary/research/	Brainsight by Rogue Research, a commercial software and hardware system for frameless stereotaxy
Cortexplore	https://www.cortexplore.com/	cortEXplore, a commercial software and hardware system for frameless and markerless planning and navigation, including real-time Augmented Reality support for surgeons
Kopf MRI stereotaxic instrument	https://kopfinstruments.com/product/model-1430m-mri-stereotaxic-instrument/	Commercial MRI-compatible (plastic) stereotaxic instrument from Kopf

## Box 1 Targeting brain regions for perturbation.

The success and the interpretation of results obtained with local perturbation techniques crucially depend on accurate targeting. Even non-invasive techniques such as FUS require precise targeting. Generally, this is a two- or three-stage process, depending on whether direct access to the brain is required. First, if implants such as recording chambers or cannulae are needed to allow access to the brain, these are generally planned using a pre-surgical MRI scan (or a combination of MRI and CT for better bone visualization). Several software packages are available to aid pre-surgical planning (Daye et al., 2013; Miocinovic et al., 2007 ; Ohayon and Tsao, 2012 Sperka and Ditterich, 2011) (Table 1; Fig. B1A). Next, the positioning of implants is confirmed with a post-surgical scan, the planned penetration trajectories are adjusted, and an experiment with or without a concurrent visualization is performed.

Visualization of electrodes and injections. Within-session visualization is crucial for microstimulation and local injection approaches. Microstimulation can be performed with a chronically implanted microwire array (Premereur et al., 2013 – in which case it needs to be visualized only once – or acutely with microelectrodes that are inserted in each session. If the electrode itself is “MRI-compatible” (e.g., made from a Pt-Ir alloy), the magnetic susceptibility imaging artifact is small enough so that the advancement and the final electrode position can be quickly assessed by a T2-weighted scan with high in-plane resolution (0.25 mm) and a slice along the electrode track (Fig. B1B). The susceptibility artifact can be minimized by disconnecting the leads from the electrode during the anatomical image acquisition. Such T2-weighted scans can then be co-registered to a high-resolution T1-weighted scan, done with or without an electrode in place. Similarly, injections (e.g., for reversible inactivation or viral injections for optogenetics) can be delivered via an implanted MRI-compatible external cannula (made out of PEEK or fused silica, e.g. from http://www.invivo1.com, formerly Plastics One) through which the internal cannula can be inserted, or acutely via a craniotomy, stereotactically or using a recording chamber, typically with a grid and a guide tube. The pharmacological and viral injection site and substance spread can be assessed with co-injection of an MRI contrast agent. Paramagnetic substances based on Gadolinium (Gd) (Heiss et al., 2010 Yazdan-Shahmorad et al., 2016), such as Gadovist or Dotarem, and Manganese (Mn) (Chen et al., 2016; Fredericks et al., 2020 are typically used. Mn is also used for in vivo visualization of trans-synaptic spread (Chen et al., 2019). In the future, substances that combine the neural perturbation action and have intrinsic MRI visibility, such as a newly developed paramagnetic analog of muscimol, might be utilized (Bricault et al., 2020).

Stereotaxic alignment. Since most implantation surgeries are performed in a stereotaxic plane, the alignment of the pre-surgical MRI into a stereotaxic space (also known as the Frankfurt horizontal baseline line, joining the inferior orbital margin and the bony external auditory meatus, i.e. the ear canal) is of utmost importance, especially when targeting deeper structures (for a subcortical atlas in stereotaxic space, see Hartig et al., 2021). While identifying the ear canal and the corresponding interaural line is relatively easy, the location of the inferior orbital margin is often harder to estimate based on the MR image alone. This could lead to an image plane tilted with respect to the true stereotaxic plane by several degrees, and lead to a considerable offset between intended and implanted locations. Therefore, scanning while the animal is positioned in the MRI-compatible plastic stereotaxic holder with integrated MRI-visible markers (e.g., Kopf model 1430 M) is preferable, although even in this case caution should be exercised to check and minimize a residual flex of the ear bars and infra-orbital clamps. Additional landmarks (e.g. mid ocular plane, temporomandibular joint) can also be useful for alignment. In lieu of scanning in an MRI-compatible stereotaxic holder, an MRI can be placed in the stereotaxic orientation by alignment (e.g., using AFNI’s @animal_warper) to a stereotaxically aligned template, such as the macaque NMT v2 (Jung et al., 2021). Note that the commonly used AC-PC plane (the horizontal line connecting anterior and posterior commissures, or passing above AC and below PC) is only a rough proxy for the true stereotaxic plane because in macaques the AC-PC plane is typically slightly tilted (roughly 3-15° “nose downward”) relative to former. Frameless stereotaxy. An alternative or complementary method to pre-surgical stereotaxic planning is a frameless stereotaxy that relies on the online coregistration of fiducial markers visible on the scan and in the real physical space, e.g. embedded in the headcap or rigidly attached to the animal’s head post (Frey et al., 2004). Neuronavigation systems employ an optical position sensor to enable tracking of the animal’s head and the localization of optimal paths towards potential targets using real time display software. The main advantages of this approach are its flexibility (e.g. the animal does not have to be placed into a stereotaxic holder) and the possibility for online readjustments, but it requires a dedicated hardware solution (e.g. BrainSight).

MRI-guided navigation. Several software packages offer an intermediate solution allowing detailed pre-surgical planning, post-surgical visualization, and online updating of coordinates and angles without additional hardware requirements. Planner (Ohayon and Tsao, 2012) relies on registering a set of external fiducial markers visible in the MRI scan to a set of measurements obtained during the surgery. As long as a model of a stereotaxic manipulator arm is provided and it is rigidly attached relative to the skull (not necessarily using a stereotaxic holder), it solves the registration problem and outputs a set of parameters needed to position the manipulator to reach a specified brain site along arbitrary, non-vertical trajectories. Cicerone (Miocinovic et al., 2007) also enables real-time interactive visualization of the electrode location in 3D relative to the surrounding neuroanatomy and neurophysiology, using stereotaxic microdrive coordinates.

Vessel visualization. Proper targeting alleviates the risks of rupturing a blood vessel during electrode or cannula insertion and, in the case of GABA-A inactivations, of inadvertent injection of the drug into a ventricle, which can cause respiratory suppression. Major blood vessels can be delineated from a standard T1-w scan, for instance with the CIVET-macaque software (Lepage et al., 2021). Vessel visualization can be aided by a T1-w scan with a Gd contrast agent bolus injected intravenously prior to the scan, a co-registered T2-w or DWI scan that accentuates blood vessels, and the use of a local transmit-receive coil rather than a receive-only coil together with the integrated body transmit coil. The Planner software provides built-in functionality for vessel detection, visualization, and avoidance using optimal trajectory planning.

DBS electrode visualization. For the post-surgical localization of implanted intracranial electrodes, a new toolbox has been developed through a close collaboration between NHP researchers and the developers of Lead-DBS (a Matlab toolbox for localizing DBS leads in patients (Horn et al., 2017)) that can accurately localize DBS leads in macaque monkeys. This open-source toolbox is a big step towards better localization of DBS leads and understanding of clinical effects through connectomics. The LEAD-DBS algorithm offers several methods to preprocess the data based on pre- and post-operative MRI and/or CT scans, but it is flexible enough to accept images previously corrected by custom-made tools. The main DBS electrode references from common manufacturers (e.g., Medtronic, Abbott-St Jude, Boston Scientific) are included in the package as models and new geometry specifications can be implemented. LEAD-DBS macaque reconstructs the electrode trajectory to determine the exact placement of each contact and computes the Volume of Activated Tissue (VAT) based on four distinct physical models. The VAT simulation, which is only available for the clinical DBS electrodes, is of interest to identify the cerebral nuclei where the electrical stimulation is delivered, in relationship to an atlas, functional ROIs or fiber-tracking connectomes. The anatomical location of the DBS leads can be linked to functional and diffusion data to ultimately facilitate the study of behavioral effects. The current limitation of this tool is that its best features are restricted to clinical leads only.

## Glossary

### Abbreviations

Antidromic effect: An effect caused by propagation of impulses against the direction of normal neuronal information transfer, i.e. traveling an axon away from the terminals towards the soma.
Blood-brain-barrier (BBB): The microvasculature of the brain forms a semi-permeable barrier that allows the passage of some substrates but blocks others. The characteristics of the BBB can be altered by some perturbation methods such as focused ultrasound stimulation in combination with micro-bubbles.
Compensatory activity/plasticity: Neural activity/plasticity that occurs as a consequence of brain perturbations and appears to be related to the normalization of the brain processes that are affected by the perturbation.
Deep Brain Stimulation (DBS): Stimulation of subcortical brain areas. Clinical DBS is done with electrical stimulation through relatively large macro-electrodes. In experimental neuroscience, DBS can also be performed with micro-electrodes.
DREADDs: Designer Receptors Exclusively Activated by Designer Drugs (DREADDs) are a class of genetically engineered G-protein coupled receptors that are chemokinetically activated by a synthetic ligand such as CNO (Clozapine N-oxide).
Effective connectivity: Stimulation-evoked activity modulations in regions that are not directly stimulated, e.g. if stimulation of region A evokes neural activity in distinct region B, regions A and B are thought to be effectively connected.
Electrical stimulation: Stimulation with electric current through some type of stimulation electrode.
Focused ultrasound stimulation: Non-invasive brain perturbation technique based on pulsed focused ultrasound delivered to the brain with a ceramic transducer.
Functional connectivity: Correlated activity. The term is commonly used to illustrate correlated activity patterns, often in resting state neural data.
Infrared neuromodulation: Optical stimulation method that employs long wavelength laser light to focally stimulate the brain via brief pulses of optically induced heat transients.
Lesions (irreversible/reversible): A lesion is any damage to biological tissue or functional disruption that can occur naturally or be experimentally evoked. We dissociate permanent, irreversible lesions such as those resulting from axonal transection (cutting of nerve fibers), aspiration (removal of tissue), or cytotoxic injections, from reversible pharmacological lesions that only temporarily deactivate a brain area.
Microstimulation: Spatially localized electrical stimulation with microelectrodes.
Microbubbles: An intravenous injection of lipid microbubbles can, in conjunction with focused ultrasound sonication, make the blood-brain-barrier permeable for substrates that would otherwise be blocked.
Monosynaptic effect: A perturbation effect that is observed in neurons or brain areas that are directly connected to the site of perturbation via a single synapse.
Optogenetic stimulation: A genetic method to express lightsensitive ion channels in specific populations of neurons. This allows the activation or suppression of these neurons with light of construct-specific wavelengths.
Orthodromic effect: An effect caused by propagation of impulses along the direction of normal neuronal information transfer, i.e. traveling an axon away from the soma towards the terminals.
Polysynaptic effect: A perturbation effect that is observed in neurons or brain areas that are only indirectly connected to the site of perturbation via multiple synaptic connections.
Resting state: Brain activity in the absence of a task, i.e. with the subject ‘at rest’ or during simple eye fixation in the absence of stimuli.
Structural connectivity: Physical connection through axonal projections spanning one or more synapses.

## Footnotes

1
https://www.nimh.nih.gov/research/research-funded-by-nimh/therapeutics/cns-radiotracer-table.shtml

2 Connectors void of nickel can be obtained from Omnetics at special request (part numbers: Ni-Free A9670-001, and Ni-Free A9671-001).

3
https://www.prizmatix.com/UHP/UHPLEDs.aspx
